# The role of smoking and alcohol in mediating the effect of gastroesophageal reflux disease on lung cancer: A Mendelian randomization study

**DOI:** 10.3389/fgene.2022.1054132

**Published:** 2023-01-16

**Authors:** Jing Yang, Duorui Nie, Yujing Chen, Zixing Liu, Mengzhao Li, Chun Gong, Qiong Liu

**Affiliations:** ^1^ The First Clinical Medical School of Guangzhou University of Chinese Medicine, Guangzhou, China; ^2^ Graduate school of Hunan University of Chinese Medicine, Changsha, China; ^3^ The First Affiliated Hospital, Hunan University of Chinese Medicine, Changsha, Hunan, China; ^4^ The First Affiliated Hospital, Guangzhou University of Chinese Medicine, Guangzhou, Guangdong, China

**Keywords:** lung cancer, gastroesophageal reflux disease, prospective analysis, causality, Mendelian randomization study

## Abstract

Observational studies have suggested a positive association between gastroesophageal reflux disease and lung cancer, but due to the existence of confounders, it remains undetermined whether gastroesophageal reflux disease (GERD) has a causal association with lung cancer. Therefore, Mendelian randomization (MR) analyses were applied to investigate the relationship between the two conditions. Two-sample Mendelian randomization analysis was utilized with summary genetic data from the European Bioinformatics Institute (602,604 individuals) and International Lung Cancer Consortium, which provides information on lung cancer and its histological subgroups. Furthermore, we used two-step Mendelian randomization and multivariable Mendelian randomization to estimate whether smoking initiation (311,629 cases and 321,173 controls) and alcohol intake frequency (*n* = 462,346) mediate any effect of gastroesophageal reflux disease on lung cancer risk. The Mendelian randomization analyses indicated that gastroesophageal reflux disease was associated with and significantly increased the risk of lung cancer (OR_IVW_ = 1.35, 95% CI = 1.18–1.54; *p* = 1.36 × 10^–5^). Smoking initiation and alcohol intake frequency mediated 35% and 3% of the total effect of gastroesophageal reflux disease on lung cancer, respectively. The combined effect of these two factors accounted for 60% of the total effect. In conclusion, gastroesophageal reflux disease is associated with an increased risk of lung cancer, and interventions to reduce smoking and alcohol intake may reduce the incidence of lung cancer.

## 1 Introduction

According to the 2020 global cancer statistics analysis, lung cancer is the second most common cancer with the highest mortality rate (Sung, et al., 2021) and an estimated 1.8 million deaths. Since targeted prevention and early screening might help reduce the morbidity and clinical burden (O'Neil, et al., 2019), it is important to determine the other underlying pathogenic factors, for instance, smoking, occupational exposure, history of non-infectious-related respiratory diseases, and gastroesophageal reflux disease (GERD) (Pallis and Syrigos, 2013; Herbella, et al., 2015; Schabath and Cote, 2019).

However, in comparison with other risk factors, there have been few studies that reported the relationship between GERD and lung cancer. GERD is a widespread and chronic ailment, and the incidence has increased to approximately 13% over the past few decades (Richter and Rubenstein, 2018; GBD 2017 Oesophageal Cancer Collaborators, 2020). The most frequent presentation of GERD includes heartburn, acid regurgitation, and extra-esophageal symptoms, which occur weekly at least. GERD is an important risk factor for chronic respiratory disorders, such as chronic cough, asthma, and other complications because of the recurrent acid stimulation (Hungin, et al., 2005; Chung and Pavord, 2008; Pacheco-Galván, et al., 2011; Solidoro, et al., 2017). Furthermore, there are some studies proposing an association between GERD and lung cancer (Hsu, et al., 2016; Yanes, et al., 2020). Vereczkei et al. (2008) found that the ratio of diagnosed gastroesophageal reflux cases in lung cancer was comparatively higher than the average population, but this study only involved 25 patients with lung cancer and did not prove whether there exists a causative connection between GERD and lung cancer. One large cohort study has proved that the additional risk of lung cancer may be due to GERD in Asians (Hsu, et al., 2016). However, the association between GERD and lung cancer in the European population remains unclear. The effect of GERD on lung cancer risk may in part be mediated by common risk factors such as BMI, smoking, and alcohol (Malhotra, et al., 2016).

Observational studies cannot systematically evaluate the relationship between the two diseases because there may be biases such as confounders or reverse causality. Using Mendelian randomization (MR) can eliminate the effect of confounders and avoid reverse causation and various errors commonly found in observational epidemiological studies (Ebrahim and Davey Smith, 2008). MR selects instrumental exposure variables to prove the causation of the risk factors relevant to certain conditions (Smith and Ebrahim, 2003). The genetic variants should match the following conditions (Davey Smith and Hemani, 2014): first, the instrumental variable should be associated with GERD; second, the instrumental variable should not have a connection with confounders; third, the instrumental variable should not directly affect lung cancer but only through exposure. Also, horizontal pleiotropy should be ruled out.

This study employed two-sample Mendelian randomization to reveal the causation between GERD and lung cancer. Furthermore, we used two-step MR and multivariable MR to investigate whether these common risk factors mediate the effects of GERD.

## 2 Materials and methods

Genome-wide association study (GWAS) summary data were obtained from publicly available GWAS consortia. Since the analyses were conducted with published studies and public databases, no ethics approval was required.

### 2.1 GWAS exposure dataset

GERD was extracted from a GWAS consisting of 602,604 individuals of European ancestry from the European Bioinformatics Institute (EBI) (Ong, et al., 2022) available in the GWAS Catalog (https://www.ebi.ac.uk/gwas/) (Buniello, et al., 2019). Single-nucleotide polymorphisms (SNPs) among the genome-wide significant SNPs (*p* < 5 × 10^–8^) were identified in the gastroesophageal reflux disease GWAS. The genetic variants with ambiguous strand codification (A/T or C/G) were removed. Finally, we excluded the relevant SNPs based on a linkage disequilibrium level of *r* ^2^ < 0.001 and LD distance >10,000 kb. Next, F-statistics (Palmer, et al., 2012) were calculated to evaluate the strength of instrumental variables, which is relevant to the explained variance of exposure (*R2*), sample size (*n*), and the number of SNPs (*k*) according to the following formula: $F=\left[\left(n-k-1\right)/k\right]/\left[R2/\left(1-R2\right)\right]$ (Burgess and Thompson, 2011). In general, F > 10 demonstrates that SNPs could predict exposures well. In total, 80 SNPs were identified as robustly associated with GERD, and the F-statistic value for individual SNPs ranged from 208 to 669, illustrating that those SNPs are strong instrumental variables. These SNPs were used as instrumental variables to assess the effect of GERD on lung cancer and subgroups (Supplementary Table S1). SNPs rs2145318, rs957345, and rs2358016 were removed from the 80 SNPs for being palindromic with intermediate allele frequency.

Smoking initiation was extracted from the GWAS & Sequencing Consortium of Alcohol and Nicotine use of 607,291 sample size. Alcohol intake frequency was obtained from the MRC-IEU consortium consisting of 462,346 participants. All participants were of European ancestry without discrimination of sex. The SNPs of potential mediators are presented in Supplementary Table S1.

The SNPs of lung cancer were obtained from a GWAS meta-analysis, which combined their OncoArray results (McKay, et al., 2017) with the previous lung cancer GWAS (Timofeeva, et al., 2012; Wang, et al., 2014; Wang, et al., 2015). A total of 85,716 individuals (29,266 lung cancer cases and 56,450 controls) were extracted from the International Lung Cancer Consortium (TRICL-ILCCO) and Lung Cancer Cohort Consortium (LC3). They also supplied detailed statistics of lung cancer histological types (adenocarcinoma, squamous cell cancer, and small cell cancer), and there were 11,273 adenocarcinoma cases, 7,426 squamous cell cancer cases, 2,664 small cell cancer cases, and the remaining 27% contained other histological subtypes, such as large cell carcinoma, non-small cell lung cancer, NOS, mixed histology, and unknown.

### 2.2 Statistical analysis

The principal method of two-sample MR conducted in this study was inverse variance weighting (IVW) (Ehret, et al., 2011), followed by MR-Egger and weighted median (Bowden, et al., 2016). Compared to that from IVW, the standard error of the causal estimate from the MR-Egger method will typically be large and the causal estimates will be low (Burgess and Thompson, 2017). Therefore, IVW was utilized to illustrate the causality between exposures and outcomes (Burgess, et al., 2015), and the results were shown as odds ratios (ORs) and 95% confidence intervals (CIs). Three sensitivity analyses were conducted based on distinct and contrasting assumptions, weighted median, and MR-Egger. MR-Egger is used to investigate the potential bias introduced by pleiotropy (Bowden, et al., 2015) and also provides an intercept test to determine whether there is an unbiased estimate of the causal effect (Burgess and Thompson, 2017). The weighted median analysis calculates the median of an empirical distribution of MR association estimates weighted for their accuracy, providing consistent estimates when more than half of the instruments are valid (Bowden, et al., 2016).

Several sensitivity tests were utilized, including Cochran’s Q-statistic to estimate heterogeneity and MR Pleiotropy RESidual Sum and Outlier methods (MR-PRESSO) to evaluate and rectify horizontal pleiotropy (Ong and MacGregor, 2019). Compared to IVW and MR-Egger, MR-PRESSO is less biased and has better precision when the number of horizontal pleiotropy variants is lower than 10% (Verbanck, et al., 2018). Leave-one-out analysis was employed to evaluate whether a single SNP drove or influenced the MR results. Finally, the online web tool (http://glimmer.rstudio.com/kn3in/mRnd/) was used to calculate statistical power (Brion, et al., 2013).

**FIGURE 1:**
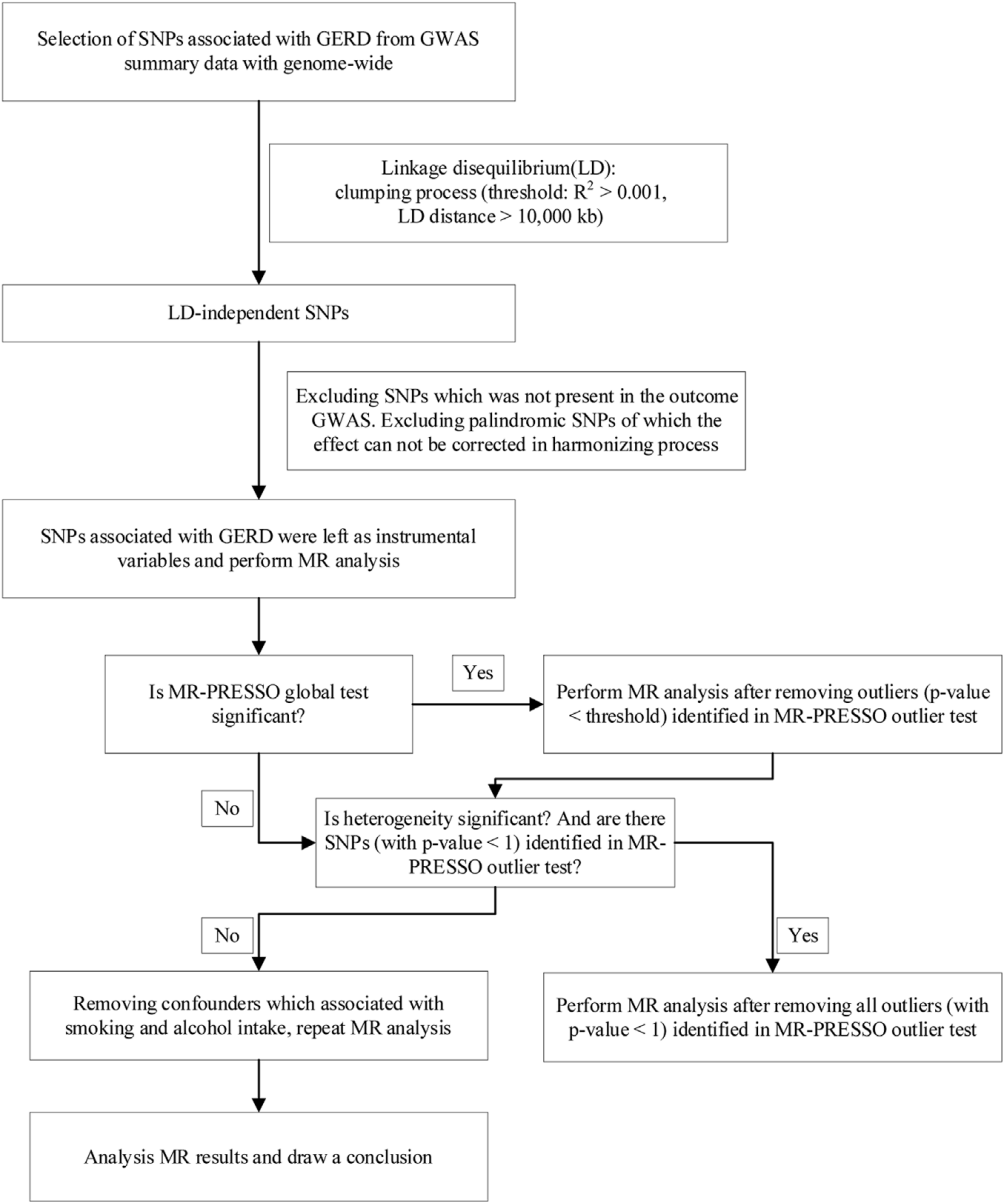
Flow chart for the analytical methods and how MR analysis was performed step-by-step.

In addition, considering smoking and alcohol as potential confounders of lung cancer, we extracted genetic instruments from the PhenoScanner GWAS database (http://www.phenoscanner.medschl.cam.ac.uk/) to obtain SNPs of smoking and alcohol intake (*p* < 5 × 10^–8^). The detailed selection of instrumental variables (IVs) and MR analysis steps are presented in Figure 1.

**FIGURE 2:**
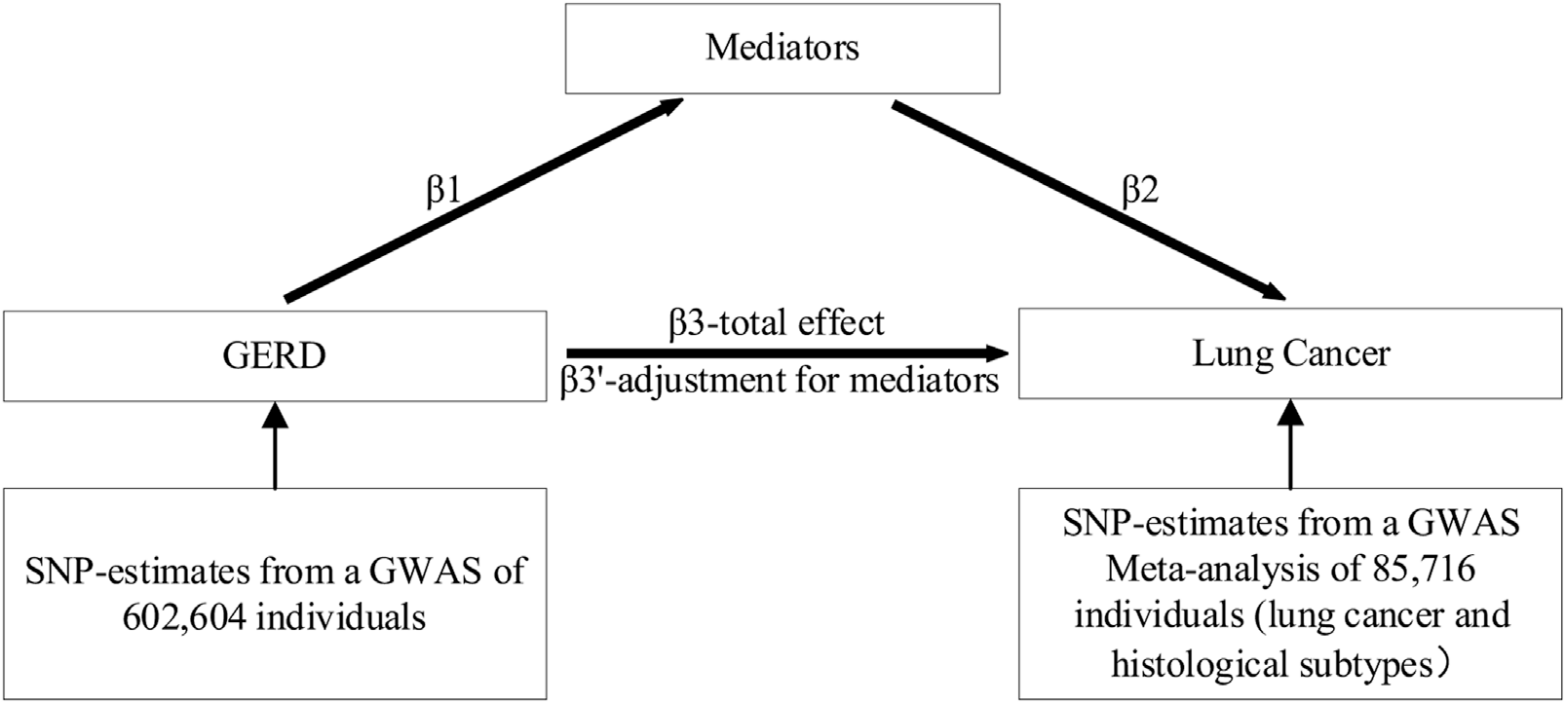
Graphical representation of the proposed mediation through mediators for the association of GERD with lung cancer. β1 represents the regression coefficients for the association between GERD and mediators; β2 represents the regression coefficients for the association between mediators and lung cancer; β3 represents the total effect between GERD and lung cancer, without the adjustment for mediators; and β3’ represents the direct effect between GERD and lung cancer, taking into account the adjustment for mediators.

To estimate and quantify the effects of mediators, we used two-step MR and multivariable MR (MVMR). The two-step approach is considerably less prone to the biases inherent in the common multivariable approach (Richmond, et al., 2016). In MVMR, the total effect of each exposure is decomposed into direct and indirect effects. A graphical depiction of the analyses is shown in Figure 2. As shown in Figure 2, mediation was considered to be present when meeting the following conditions: 1) GERD was correlated with mediators (β1); 2) GERD was associated with lung cancer without adjustment for mediators (β3); 3) mediators were associated with lung cancer (β2); and 4) the association of GERD with lung cancer decreased upon the addition of mediators to the MVMR model (β3’). The percentage of mediation is calculated using the following formula: proportion mediated = $\left(\beta 1\times \beta 2\right)/\left(\beta 3\right)$ .

MR analysis was conducted using TwoSampleMR (version 0.5.6) in the R package (version 4.2.1). The Bonferroni correction was employed to counteract the problem of multiple comparisons because three histological types were considered (*p* = .05/4 = .0125) (Armstrong, 2014).

## 3 Results

The F-statistics for all genetic instruments were >10, indicating that IVW analyses were unlikely subjected to weak-instrument bias. If the power is greater than 0.8, statistical power is considered adequate. The statistical power result is presented in Supplementary Table S2, indicating that our study was adequate for both statistical significance and statistical power.

**FIGURE 3:**
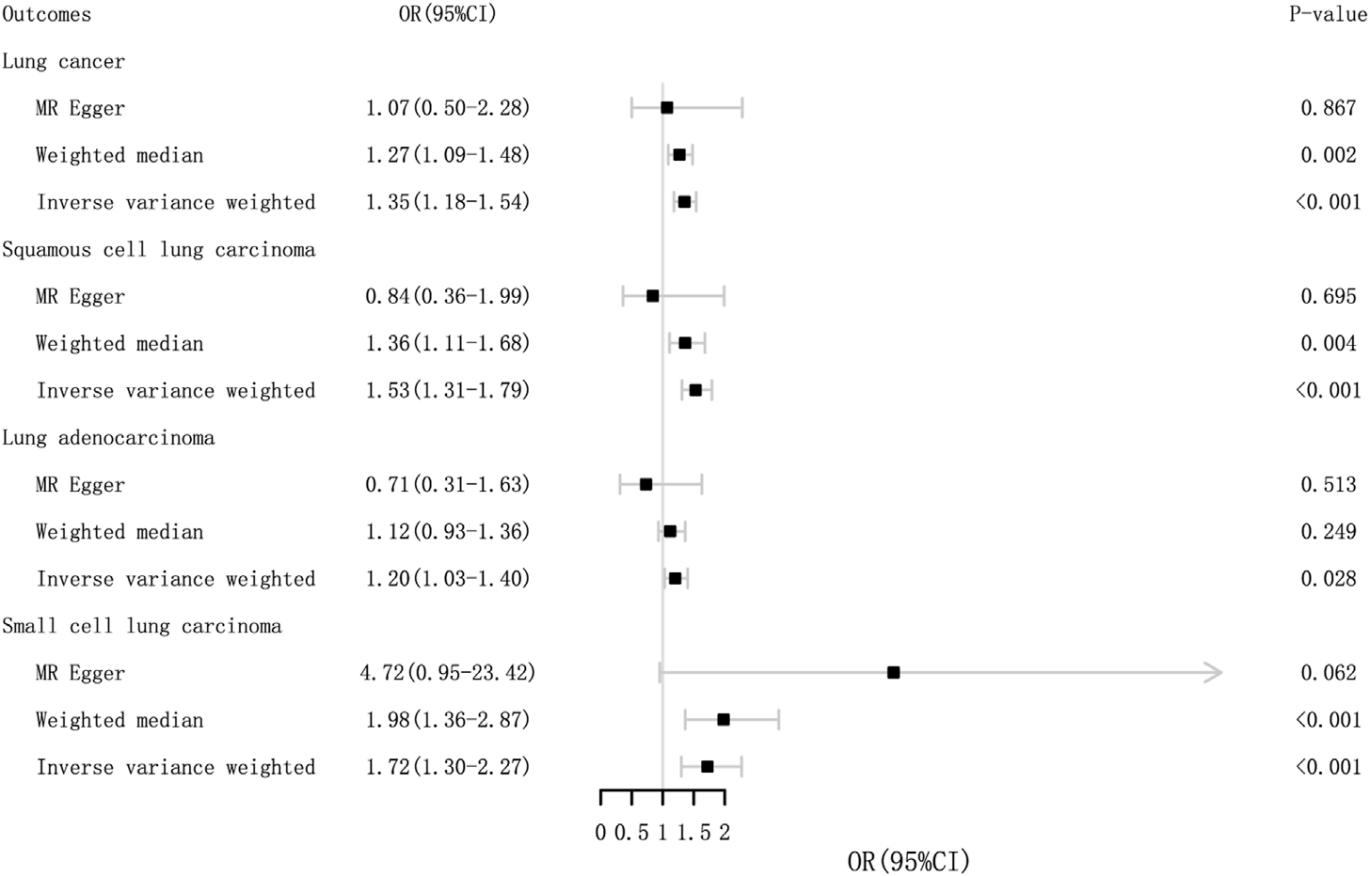
Odds ratios and *p*-value of MR analysis for the associations between GERD and lung cancer in the EBI. OR, odds ratio; 95% CI, 95% confidence interval.

MR analysis indicated that GERD was associated with and significantly increased the risk of lung cancer (OR_IVW_ = 1.40, 95% CI = 1.18–1.54; *p* = 1.36 × 10^–5^). Meanwhile, similar sensitivity analyses were obtained using MR-Egger (OR = 1.07, 95% CI = .50–2.28; *p* = 0.867) and weighted median methods (OR = 1.27, 95% CI = 1.09–1.48; *p* = .002). There was also a causal relationship between GERD and squamous cell cancer and small cell cancer. The MR results are presented in Table 1 and Figure 3. There was no pleiotropy, with IVW and MR-Egger reflecting unbiased estimates for causality. For some associations, the causality from weighted median MR and MR-Egger is broadly consistent with that from IVW MR, although with wider confidence intervals due to lower statistical power (Verbanck, et al., 2018). For instance, the causal estimates of GERD on squamous cell lung cancer were consistent in IVW MR (OR = 1.53, 95% CI = 1.31–1.79; *p* = 7.84 × 10^–8^) and weighted median MR (OR = 1.36, 95% CI = 1.11–1.68; *p* = .004).

**TABLE 1:** MR estimates from different methods of assessing the causal effect of GERD on lung cancer and subtypes.

Outcome	Step	No. of SNPs	IVW OR (95% CI)	*p*-value	Cochran’s Q-statistics	Q-pval	Weighted median OR (95% CI)	*p*-value	MR-Egger OR (95% CI)	*p*-value	Cochran’s Q-statistics	Q-pval	Egger-intercept	Intercept (Se)	*p*-value	MR-PRESSO global test *p*-value	MR-PRESSO distortion test *p*-value
Lung cancer	1	75	1.40 (1.18–1.54)	1.36E-05	170.79	1.28E-09	1.27 (1.09–1.48)	.002	1.07 (.50–2.28)	.867	169.92	1.06E-09	.008	.013	.541	<2e-04	.749
	2	72	1.38 (1.22–1.56)	1.83E-07	126.50	5.67E-05	1.27 (1.10–1.47)	.001	1.00 (.51–1.96)	.996	124.89	6.06E-05	.011	.011	.346	<5e-04	NA
	3	68	1.29 (1.16–1.44)	6.99E-06	95.63	.012	1.25 (1.08–1.44)	.002	1.00 (.54–1.83)	.990	94.60	.012	.009	.010	.398	.011	NA
	4	60	1.28 (1.15–1.44)	1.92E-05	77.15	.056	1.25 (1.08–1.45)	.003	.74 (.38–1.43)	.368	73.61	.081	.018	.011	.100	.074	NA
Squamous cell lung carcinoma	1	75	1.53 (1.31–1.79)	7.84E-08	88.49	.120	1.36 (1.11–1.68)	.004	.84 (.36–1.99)	.695	86.19	.139	.020	.014	.168	.142	NA
	4	64	1.51 (1.27–1.80)	2.30E-06	74.35	.155	1.40 (1.11–1.77)	.005	0.79 (0.27–2.28)	.660	72.61	.168	.021	.017	.227	.176	NA
Lung adenocarcinoma	1	73	1.21 (1.02–1.43)	.028	129.40	3.90E-05	1.12 (1.02–1.43)	.240	.73 (.29–1.85)	.513	127.33	4.66E-05	.016	.015	.286	<2e-04	0.949
	2	71	1.20 (1.03–1.40)	.019	102.39	.007	1.12 (0.93–1.36)	.237	.71 (.31–1.63)	.424	100.09	.009	.017	.014	.213	.008	NA
	3	68	1.22 (1.06–1.41)	.006	82.71	.093	1.22 (0.96–1.42)	.128	.69 (.32–1.48)	.340	79.97	.116	.019	.012	.137	.094	NA
	4	58	1.17 (1.00–1.37)	.051	67.57	.160	1.06 (0.86–1.30)	.606	.38 (.15–0.95)	.044	61.14	.297	.037	.150	.019	.169	NA
Small cell lung carcinoma	1	67	1.72 (1.30–2.27)	1.69E-04	92.51	.017	1.98 (1.36–2.87)	3.29E-04	4.72 (.95–23.42)	.062	90.32	.021	-.034	.027	.213	.023	NA
	2	66	1.79 (1.36–2.35)	2.78E-05	86.77	.074	1.99 (1.40–2.83)	1.33E-04	6.08 (1.30–28.34)	.025	81.02	.074	-.041	.257	.119	.074	NA
	4	56	1.92 (1.28–2.90)	2.28E-04	73.27	.050	1.92 (1.28–2.90)	.002	4.31 (0.54–34.26)	.173	72.31	.049	-.028	.034	.403	.056	NA

Step 1, MR analysis with the complete selected SNPs; step 2, MR analysis after removing the SNPs (with a *p*-value less than the threshold in the MR-PRESSO outlier test); step 3, MR analysis after removing all the SNPs (with a *p*-value less than 1 in the MR-PRESSO outlier test); step 4, on the basis of step 3, MR analysis after removing all confounders (associated with smoking and alcohol intake).

Regarding lung cancer, the MR-Egger intercept tests presented no statistically significant horizontal pleiotropy (*p* intercept = .541), but Cochran’s Q test indicated significant heterogeneity in estimating individual SNPs (*p* = 1.28 × 10^–9^). MR-PRESSO also revealed a similar result (*p*-value in the global heterogeneity test <0.001). The sensitivity analyses demonstrated heterogeneity in lung cancer and adenocarcinoma, without heterogeneity observed in the other subgroup MR results. The detailed sensitivity analysis results are presented in Table 1. The MR-PRESSO distortion test was employed to evaluate the causal estimate from IVW before and after the removal of the horizontal pleiotropic outlier variants because of the existence of heterogeneity. The *p*-value of the MR-PRESSO distortion test in lung cancer and lung adenocarcinoma indicated that the horizontal pleiotropic outlier variants did not distort the causal estimate (Verbanck, et al., 2018).

No single SNP strongly violated the overall effect of GERD on lung cancer and the subgroups in the leave-one-out sensitivity analysis. Furthermore, the funnel plot was approximately symmetrical, indicating no pleiotropy. All results are presented in Supplementary Figure S1.

After ruling out outliers in lung cancer and subgroups, the two-sample MR analysis was re-applied to evaluate the causation. The outliers are presented in Supplementary Table S3. Furthermore, we found eleven genetic instruments which are associated with smoking and alcohol intake (Supplementary Table S4). After removing these confounders, similar causalities were presented in all groups. As shown in Figure 1, MR analysis was repeated multiple times until the heterogeneity was removed. These results suggest a strong causal effect between GERD and lung cancer.

In univariable MR, we observed evidence of the association between GERD and the two mediators, and both of them were correlated with lung cancer and squamous cell lung carcinoma (Table 2). The MR results supported an association between smoking initiation and small cell lung cancer. In MVMR analysis, the effect of GERD showed different degrees of decline in lung cancer and squamous cell lung carcinoma. However, in small cell lung carcinoma, only smoking initiation fulfilled the criteria for mediation. The results are presented in Table 3.

**TABLE 2:** Two-step MR estimates of GERD on mediators and mediators on lung cancer.

Exposure	Outcome	Method	No. of SNPs	OR (95% CI)	*p*-value
GERD	Smoking initiation	MR-Egger	5	.59 (.25–1.39)	.314
		Weighted median	5	2.07 (1.74–2.46)	2.01E-16
		Inverse variance weighted	5	2.16 (1.85–2.52)	2.17E-22
GERD	Alcohol intake frequency	MR-Egger	9	1.44 (1.09–1.91)	.037
		Weighted median	9	1.77 (1.60–1.96)	2.86E-28
		Inverse variance weighted	9	1.75 (1.62–1.90)	4.23E-43
Smoking initiation	Lung cancer	MR-Egger	71	1.67 (.83–3.36)	.155
		Weighted median	71	1.72 (1.44–2.06)	3.72E-09
		Inverse variance weighted	71	1.68 (1.47–1.92)	6.30E-14
Alcohol intake frequency	Lung cancer	MR-Egger	79	.87 (.50–1.51)	.624
		Weighted median	79	.89 (.73–1.11)	.109
		Inverse variance weighted	79	1.06 (.91–1.24)	.079
Smoking initiation	Small cell lung carcinoma	MR-Egger	79	1.78 (.43–7.45)	.432
		Weighted median	79	1.54 (1.01–2.35)	.043
		Inverse variance weighted	79	1.81 (1.37–2.40)	3.49E-05
Alcohol intake frequency	Small cell lung carcinoma	MR-Egger	82	.68 (.38–1.21)	.194
		Weighted median	82	.84 (.51–1.38)	.489
		Inverse variance weighted	82	1.15 (.84–1.57)	.377
Smoking initiation	Squamous cell lung carcinoma	MR-Egger	78	2.39 (0.82–6.97)	.114
		Weighted median	78	1.86 (1.41–2.47)	1.25E-05
		Inverse variance weighted	78	2.05 (1.67–2.51)	5.18E-12
Alcohol intake frequency	Squamous cell lung carcinoma	MR-Egger	90	.95 (.45–2.01)	.895
		Weighted median	90	1.23 (.91–1.67)	.178
		Inverse variance weighted	90	1.36 (1.10–1.68)	.004

**TABLE 3:** Multivariate separate-sample MR analysis of the effect of GERD on lung cancer and subgroups.

	Outcome	OR	95% CI	*p*-value	Mediation effect (%)
Univariable MR-IVW for GERD	Lung cancer	1.28	(1.15–1.44)	1.92E-05	
	Lung adenocarcinoma	1.17	(1.00–1.37)	.051	
	Small cell lung carcinoma	1.79	(1.36–2.35)	2.78E-05	
	Squamous cell lung carcinoma	1.51	(1.27–1.80)	2.30E-06	
Multivariate model					
(1) Adjusted for smoking initiation	Lung cancer				35
(2) Adjusted for alcohol intake frequency	Lung cancer	1.17	(1.02–1.34)	.024	3
(3) Adjusted for smoking initiation + alcohol intake frequency	Lung cancer	1.13	(.95–1.33)	.162	60
(4) Adjusted for smoking initiation	Small cell lung carcinoma	1.53	(1.15–2.02)	.003	30.8
(5) Adjusted for smoking initiation	Squamous cell lung carcinoma	1.26	(1.04–1.52)	.014	51.7
(6) Adjusted for alcohol intake frequency	Squamous cell lung carcinoma	1.46	(1.22–1.75)	4.42E-05	7
(7) Adjusted for smoking initiation + alcohol intake frequency	Squamous cell lung carcinoma	1.25	(.99–1.57)	.058	72

## 4 Discussion

Two-sample MR analysis was performed to assess the relationship between GERD and lung cancer, showing that GERD significantly increased the risk of lung cancer. GERD is positively associated with lung cancer in an Asian population (Hsu, et al., 2016), but the study did not consider smoking as a confounder, which is a strong risk factor for both GERD and lung cancer (Pandolfino and Kahrilas, 2000; Warren and Cummings, 2013). After ruling out the SNPs of smoking, our study identified a substantially causal association between GERD and lung cancer compared to previous studies.

Previously, all-Nordic cohort research indicated that patients undergoing an antireflux operation had a reduced risk of small cell and squamous cell tumors of the lungs (Yanes, et al., 2020) but not of lung adenocarcinoma. Surprisingly, a pilot study obtained the opposite conclusion (Vereczkei, et al., 2008) that the rate of consistent reflux symptoms was not significantly different between adenocarcinoma and squamous cell carcinoma groups. In our study, GERD had a robust causal association with small cell lung cancer and squamous cell carcinoma. These results are consistent with the pilot study which reported that the influence of GERD on lung cancer subtypes was no different. For the former all-Nordic study, the opposite conclusion may result from the strong effect of confounders.

In previous studies, smoking and alcohol have been considered strong risk factors of lung cancer. Meanwhile, we found that smoking and alcohol mediated most of the effect of GERD on lung cancer. Interventions to reduce smoking and quit alcohol may reduce the incidence of lung cancer at the population level, thus benefitting the patients and hospital alike. However, there was no evidence that alcohol mediated the GERD’s effect on small cell lung cancer. Nevertheless, the distinction in the influence of GERD on different lung cancer subtypes still needs further research.

The mechanism of GERD and lung diseases is still controversial (Broers, et al., 2018), and one of the more recognized hypotheses is the “reflux theory.” This theory holds that the contents of gastroesophageal reflux (acid, pepsin, bile acids, and pancreatic enzymes) through the esophagus subsequently lead to microaspiration in the lungs (Vaezi, 2005). Microaspiration negatively affects lung function, which is associated with the levels of bile acids (Broers, et al., 2018). Furthermore, an *in vitro* experiment has depicted that pepsin is cytotoxic to bronchial epithelial cells and may promote the release of TNF-α (Bathoorn, et al., 2011). The GERD effect on lung cancer may have a similar mechanism.

However, the specific role of GERD in the pathogenesis of lung cancer remains to be determined. There are several possible explanations: first, pepsin reflux can induce genes that correspond to an accelerating cellular proliferative state (Sung, et al., 2003; Dvorak, et al., 2011) as Johnston et al. (2012) demonstrated that pepsin exposure significantly altered the expression of 27 genes implicated in cellular proliferation. Second, salts and gastric acid lead to DNA damage or genetic mutations (Denlinger and Thompson, 2012; Samuels, et al., 2021), which further disrupt cell proliferation, and the critical genetic alterations may finally lead to tumor formation (Kaur, et al., 2000). Third, bile acids and pepsin can activate different cancer-related cellular pathways, such as epidermal growth factor receptor (EGFR), Notch, p38, and NF-κB (Merchant, et al., 2005; Jaiswal, et al., 2006; Niu, et al., 2020). For example, activation of NF-κB accelerates oncogenic mRNA and miRNA phenotypes and proliferation of mutant cells (Vageli, et al., 2021). In addition, GERD increases the risk of EGFR mutations among patients with advanced lung cancer (Choi, et al., 2019). In conclusion, there can be little doubt that the effect of GERD on lung cancer is significant.

Other chronic respiratory conditions are related to GERD, such as chronic cough and asthma (Kahrilas, et al., 2014; Gibson, et al., 2016; Solidoro, et al., 2017), and several studies have revealed the improvement in chronic cough and asthma with antacid treatment (Reichel, et al., 2008; Faruqi, et al., 2011; Shaheen, et al., 2011; Hait and McDonald, 2019). Similarly, the intensive treatment of GERD may help reduce the morbidity of lung cancer because of the causation between GERD and lung cancer. Antireflux drugs, while not providing adequate protection against airway aspiration, could theoretically decrease oncogenic inflammatory insults to the lungs, which results from the acidity of the reflux. Although antireflux surgery may better reduce the risk of lung cancer, further research is needed to demonstrate the effect of chemoprevention on patients with GERD.

Our study has several strengths. To the best of our knowledge, this is the first Mendelian randomization study to evaluate the causal relationship between GERD and lung cancer in the European population. Compared to clinical observational studies, MR analysis can also avoid the influence of reverse causality and confounders. To minimize the potential influence, we excluded smoking and drinking as confounders, which are the most related risk factors (Malhotra, et al., 2016; Nooreldeen and Bach, 2021). Lastly, our results may affect healthcare policies targeting GERD and lung cancer. Considering the high prevalence of the two conditions in the average population, revealing the causality may help in early prevention and timely intervention.

Nonetheless, this study also had several limitations. First, summary data of age, sex, and smoking propensity (cigarettes smoked per day) of participants were not available. Therefore, we cannot allow for stratified analyses by covariates. Second, our study was based in Europe, so further investigation is required to determine whether these findings can be applied to other races. Third, our study did not exclude some relevant chronic conditions, such as chronic obstructive pulmonary disease, hypertension, and diabetes. Fourth, given the insufficient data, the existence of reverse causality that lung cancer causes increased GERD or mediators cannot be completely ruled out.

In conclusion, MR analysis provides compelling evidence for the causality between GERD and lung cancer, but further studies are needed to elucidate the association between different lung cancer subtypes and the underlying mechanisms.

## Data Availability

The original contributions presented in the study are included in the article/Supplementary Material; further inquiries can be directed to the corresponding authors.

## Abbreviations

GWAS, genome-wide association study; OR, odds ratio; SNPs, single-nucleotide polymorphisms; MVMR, multivariable MR; MR-PRESSO, MR Pleiotropy RESidual Sum and Outlier; TRICL-ILCCO, International Lung Cancer Consortium; LC3, Lung Cancer Cohort Consortium; EBI, European Bioinformatics Institute; CI, confidence interval; IVW, inverse variance weighted; MR, Mendelian randomization; EGFR, epidermal growth factor receptor.
