# Supplementary material for: The role of smoking and alcohol in mediating the effect of gastroesophageal reflux disease on lung cancer: A Mendelian randomization study
Source: Front Genet. 2023 Jan 16;13:1054132. doi: 10.3389/fgene.2022.1054132 (PMC9885128; doi:10.3389/fgene.2022.1054132)
Supplement: Supplementary file 1 [file Table1.docx]

**Supplementary Table S1**. SNPs of exposure and mediators used in MR analyses.

| **Exposures** | **SNP** | **Effect Allele** | **Other Allele** | **Beta** | **SE** | **P-value** | **Affect Allele Frequency** | **R^2^** | **F** | **Overall F Statistics** |
| --- | --- | --- | --- | --- | --- | --- | --- | --- | --- | --- |
| **GERD** | rs569356 | G | A | -0.03792 | 0.00691 | 4.07E-08 | 0.140835 | 0.00035 | 209.76 | 20844.1 |
|  | rs7541875 | G | A | 0.027397 | 0.00485 | 1.61E-08 | 0.426069 | 0.00037 | 221.30 |  |
|  | rs2782641 | A | G | 0.027088 | 0.004946 | 4.33E-08 | 0.612669 | 0.00035 | 209.93 |  |
|  | rs2815749 | G | A | 0.038877 | 0.006022 | 1.07E-10 | 0.800974 | 0.00048 | 290.52 |  |
|  | rs3766823 | A | G | 0.03936 | 0.006385 | 7.09E-10 | 0.171467 | 0.00044 | 265.37 |  |
|  | rs1937450 | G | T | 0.031585 | 0.004845 | 7.07E-11 | 0.537739 | 0.00050 | 299.01 |  |
|  | rs17379561 | T | A | 0.053071 | 0.006866 | 1.08E-14 | 0.144391 | 0.00070 | 419.66 |  |
|  | rs7527682 | G | A | -0.02668 | 0.004822 | 3.13E-08 | 0.53725 | 0.00035 | 213.42 |  |
|  | rs903678 | A | G | 0.027738 | 0.005085 | 4.89E-08 | 0.339412 | 0.00035 | 207.98 |  |
|  | rs6711584 | A | G | 0.032255 | 0.00484 | 2.66E-11 | 0.452019 | 0.00052 | 310.73 |  |
|  | rs13409451 | G | A | -0.02771 | 0.004932 | 1.93E-08 | 0.392403 | 0.00037 | 220.69 |  |
|  | rs4300861 | T | C | 0.030713 | 0.004949 | 5.43E-10 | 0.38208 | 0.00045 | 268.53 |  |
|  | rs12997558 | A | G | 0.027818 | 0.005022 | 3.04E-08 | 0.358751 | 0.00036 | 214.63 |  |
|  | rs6722661 | A | G | -0.03225 | 0.005004 | 1.15E-10 | 0.364669 | 0.00048 | 290.63 |  |
|  | rs1596747 | G | A | 0.031087 | 0.004807 | 1.00E-10 | 0.494136 | 0.00048 | 291.28 |  |
|  | rs7600261 | T | C | 0.033803 | 0.005221 | 9.47E-11 | 0.306391 | 0.00049 | 292.81 |  |
|  | rs1011407 | G | A | -0.04206 | 0.007359 | 1.09E-08 | 0.121628 | 0.00038 | 227.88 |  |
|  | rs6780459 | T | A | 0.030551 | 0.005521 | 3.14E-08 | 0.746622 | 0.00035 | 212.88 |  |
|  | rs2016933 | G | C | -0.03103 | 0.005421 | 1.04E-08 | 0.730053 | 0.00038 | 228.71 |  |
|  | rs7612999 | A | G | 0.030523 | 0.005595 | 4.90E-08 | 0.245338 | 0.00034 | 207.96 |  |
|  | rs2240326 | A | G | -0.04717 | 0.004813 | 1.13E-22 | 0.473775 | 0.00111 | 669.24 |  |
|  | rs7675588 | A | C | -0.03352 | 0.005954 | 1.80E-08 | 0.794635 | 0.00037 | 221.10 |  |
|  | rs7685686 | G | A | -0.02792 | 0.004892 | 1.14E-08 | 0.422353 | 0.00038 | 229.33 |  |
|  | rs13107325 | T | C | 0.070144 | 0.009183 | 2.20E-14 | 0.074445 | 0.00068 | 408.86 |  |
|  | rs2164300 | T | C | -0.02648 | 0.004827 | 4.13E-08 | 0.523279 | 0.00035 | 210.81 |  |
|  | rs1510719 | C | T | -0.03888 | 0.004947 | 3.84E-15 | 0.383439 | 0.00071 | 431.12 |  |
|  | rs10010963 | T | C | -0.02698 | 0.004947 | 4.92E-08 | 0.616433 | 0.00034 | 207.51 |  |
|  | rs1592757 | C | G | 0.031105 | 0.005025 | 6.00E-10 | 0.355772 | 0.00044 | 267.38 |  |
|  | rs11953061 | T | C | 0.02816 | 0.005087 | 3.10E-08 | 0.338908 | 0.00036 | 214.20 |  |
|  | rs329122 | A | G | -0.02895 | 0.004884 | 3.05E-09 | 0.419631 | 0.00041 | 246.15 |  |
|  | rs2744961 | T | C | 0.029201 | 0.005015 | 5.81E-09 | 0.358437 | 0.00039 | 236.41 |  |
|  | rs12204714 | T | C | -0.02882 | 0.004994 | 7.92E-09 | 0.632223 | 0.00039 | 232.80 |  |
|  | rs9396740 | A | G | -0.03149 | 0.005559 | 1.47E-08 | 0.248794 | 0.00037 | 223.49 |  |
|  | rs9372625 | A | G | -0.03773 | 0.004954 | 2.62E-14 | 0.383042 | 0.00067 | 405.66 |  |
|  | rs3828917 | T | G | 0.067111 | 0.012005 | 2.27E-08 | 0.041826 | 0.00036 | 217.62 |  |
|  | rs9373363 | G | A | -0.03268 | 0.00556 | 4.13E-09 | 0.253631 | 0.00040 | 243.81 |  |
|  | rs4713692 | T | C | -0.02761 | 0.004986 | 3.07E-08 | 0.367808 | 0.00035 | 213.75 |  |
|  | rs11762636 | A | C | -0.05148 | 0.006256 | 1.88E-16 | 0.180282 | 0.00078 | 472.43 |  |
|  | rs2043539 | A | G | 0.027206 | 0.004865 | 2.24E-08 | 0.41866 | 0.00036 | 217.19 |  |
|  | rs2396133 | G | A | 0.029355 | 0.004818 | 1.11E-09 | 0.475329 | 0.00043 | 259.11 |  |
|  | rs2106353 | T | G | 0.036749 | 0.005725 | 1.37E-10 | 0.231451 | 0.00048 | 289.66 |  |
|  | rs2396766 | A | G | 0.032206 | 0.004819 | 2.34E-11 | 0.47308 | 0.00052 | 311.77 |  |
|  | rs215614 | A | G | -0.03285 | 0.004977 | 4.08E-11 | 0.629725 | 0.00050 | 303.48 |  |
|  | rs903959 | A | T | 0.029163 | 0.004916 | 2.99E-09 | 0.399262 | 0.00041 | 245.95 |  |
|  | rs3863241 | T | C | 0.032498 | 0.004815 | 1.49E-11 | 0.52696 | 0.00053 | 317.46 |  |
|  | rs7032155 | A | C | 0.02775 | 0.004914 | 1.63E-08 | 0.59185 | 0.00037 | 224.27 |  |
|  | rs4382592 | G | T | -0.03027 | 0.005251 | 8.20E-09 | 0.699524 | 0.00039 | 232.17 |  |
|  | rs3793577 | G | A | 0.027031 | 0.00485 | 2.49E-08 | 0.538279 | 0.00036 | 218.94 |  |
|  | rs12357321 | A | G | 0.031716 | 0.005231 | 1.33E-09 | 0.311087 | 0.00043 | 259.93 |  |
|  | rs1021363 | G | A | -0.03122 | 0.005022 | 5.10E-10 | 0.641992 | 0.00045 | 270.06 |  |
|  | rs761777 | G | A | 0.034534 | 0.005545 | 4.71E-10 | 0.254034 | 0.00045 | 272.50 |  |
|  | rs10837002 | G | C | 0.027649 | 0.005037 | 4.03E-08 | 0.35122 | 0.00035 | 210.02 |  |
|  | rs2734839 | T | C | -0.02835 | 0.004928 | 8.79E-09 | 0.606693 | 0.00038 | 231.19 |  |
|  | rs7942368 | T | C | -0.03397 | 0.005919 | 9.54E-09 | 0.214659 | 0.00039 | 234.52 |  |
|  | rs773109 | A | G | -0.03806 | 0.005102 | 8.71E-14 | 0.335269 | 0.00065 | 389.27 |  |
|  | rs324769 | T | C | -0.02677 | 0.004833 | 3.05E-08 | 0.449179 | 0.00035 | 213.77 |  |
|  | rs1479405 | T | C | 0.031484 | 0.005151 | 9.85E-10 | 0.3217 | 0.00043 | 260.80 |  |
|  | rs1716171 | T | C | 0.038398 | 0.005904 | 7.83E-11 | 0.790024 | 0.00049 | 294.92 |  |
|  | rs9542729 | G | C | -0.03632 | 0.005999 | 1.41E-09 | 0.20244 | 0.00043 | 256.79 |  |
|  | rs1334297 | A | G | -0.0388 | 0.005455 | 1.14E-12 | 0.734249 | 0.00059 | 354.21 |  |
|  | rs9529055 | A | G | 0.02666 | 0.004816 | 3.11E-08 | 0.475633 | 0.00035 | 213.72 |  |
|  | rs9517313 | C | G | 0.033114 | 0.004941 | 2.05E-11 | 0.383217 | 0.00052 | 312.53 |  |
|  | rs942065 | A | G | 0.030738 | 0.005009 | 8.45E-10 | 0.634045 | 0.00044 | 264.34 |  |
|  | rs10133111 | A | G | 0.041788 | 0.006508 | 1.35E-10 | 0.162996 | 0.00048 | 287.25 |  |
|  | rs9940128 | A | G | 0.033251 | 0.004863 | 8.06E-12 | 0.421755 | 0.00054 | 325.15 |  |
|  | rs12598916 | G | C | -0.03326 | 0.005392 | 6.87E-10 | 0.274798 | 0.00044 | 265.83 |  |
|  | rs7206608 | G | C | 0.029154 | 0.005145 | 1.46E-08 | 0.322927 | 0.00037 | 224.06 |  |
|  | rs12453010 | T | C | 0.029697 | 0.004933 | 1.75E-09 | 0.394803 | 0.00042 | 254.06 |  |
|  | rs7241572 | A | G | 0.036551 | 0.005975 | 9.49E-10 | 0.209101 | 0.00044 | 266.40 |  |
|  | rs1431196 | G | A | 0.03242 | 0.004864 | 2.65E-11 | 0.428432 | 0.00051 | 310.35 |  |
|  | rs12967855 | G | A | -0.03655 | 0.005134 | 1.09E-12 | 0.670435 | 0.00059 | 355.85 |  |
|  | rs9636202 | A | G | -0.03504 | 0.005472 | 1.51E-10 | 0.26663 | 0.00048 | 289.55 |  |
|  | rs2023878 | T | C | -0.03628 | 0.006119 | 3.04E-09 | 0.192377 | 0.00041 | 246.63 |  |
|  | rs1883842 | G | T | 0.030833 | 0.005368 | 9.27E-09 | 0.279255 | 0.00038 | 230.70 |  |
|  | rs2834005 | C | T | 0.0297 | 0.005173 | 9.42E-09 | 0.315 | 0.00038 | 229.47 |  |
|  | rs2838771 | C | G | -0.0281 | 0.005066 | 2.91E-08 | 0.646721 | 0.00036 | 217.48 |  |
|  | rs9615905 | T | C | 0.027566 | 0.004838 | 1.21E-08 | 0.458193 | 0.00038 | 227.43 |  |
| **Alcohol intake frequency** | rs780569  rs4503294  rs28787109  rs2244598  rs4417025  rs7514579  rs2717063  rs6727281  rs780094  rs13390019  rs10188314  rs4241258  rs72769229  rs17662759  rs1991083  rs473098  rs9829192  rs76082653  rs262240  rs9814516  rs7610856  rs1515591  rs1228589  rs28622224  rs13135092  rs11940694  rs362307  rs1229984  rs13102973  rs62339673  rs34811474  rs2159935  rs62305780  rs13178443  rs11750777  rs4916723  rs461599  rs56194430  rs9403297  rs9349379  rs12153855  rs9372625  rs62466318  rs2622167  rs73050128  rs6943160  rs4726481  rs9648478  rs2160935  rs34440851  rs11787216  rs2977454  rs74679146  rs489062  rs34473884  rs61873510  rs4242715  rs10792669  rs11223617  rs550942  rs11039429  rs1666658  rs12312693  rs7302200  rs28768122  rs7298932  rs58905411  rs1937522  rs7330939  rs2535911  rs186347  rs80292319  rs117799466  rs34631026  rs72787062  rs35105141  rs1421085  rs8043563  rs2411453  rs728538  rs9906502  rs8614  rs4968391  rs9912298  rs17690703  rs650558  rs1893659  rs5022348  rs2043677  rs9958320  rs2924321  rs4940926  rs838145  rs6030200  rs11700855  rs71651683 | A  T  A  C  A  C  A  T  C  C  T  T  T  C  T  T  T  T  T  T  A  G  A  T  G  G  T  C  C  A  A  A  G  T  A  C  C  T  A  G  C  A  T  A  A  C  T  A  T  T  T  G  C  A  A  T  A  G  A  T  T  C  C  A  C  G  A  G  T  T  T  C  C  T  A  T  C  C  G  G  A  A  T  C  T  T  A  T  T  C  A  C  A  A  G  T | T  C  G  T  G  A  C  C  T  T  C  C  A  T  C  C  G  C  C  G  C  T  G  C  A  A  C  T  T  C  G  G  C  C  G  A  A  C  G  A  T  G  C  G  C  T  G  G  C  C  C  C  T  G  G  G  G  A  G  C  C  T  T  G  T  A  G  A  C  C  G  T  G  C  G  C  T  G  T  T  G  C  G  A  C  C  C  C  C  T  G  T  G  G  A  C | 0.01980  0.01815  0.01781  -0.01838  -0.01884  0.01967  -0.02037  -0.02432  -0.05099  0.02961  -0.01979  0.02506  -0.02314  0.03013  -0.02239  -0.02174  0.01693  0.04643  -0.01721  -0.02511  -0.02386  0.01823  0.02107  -0.01862  0.04383  -0.04371  0.04330  -0.26171  -0.01941  0.01829  -0.02018  -0.01857  -0.04852  -0.01865  -0.02049  0.02395  -0.01919  0.02254  0.01882  -0.01935  0.02944  -0.02556  -0.02549  -0.01912  -0.02600  0.02063  0.02176  0.01686  -0.01872  -0.02268  0.02442  -0.02592  -0.03207  0.01665  -0.02036  0.02037  -0.01865  0.01743  0.02509  0.02240  -0.02356  0.01797  -0.01768  -0.01842  0.02070  -0.02372  -0.02663  0.01690  -0.02133  -0.01885  0.01795  -0.03937  -0.01967  -0.01691  -0.02819  0.02635  0.01994  0.02337  -0.03508  0.02288  0.02379  0.02478  -0.01927  0.02059  0.02503  0.02074  -0.02933  0.02026  0.02611  0.02486  -0.01951  -0.01910  0.02195  -0.01953  -0.02980  -0.07046 | 0.00336454  0.00307049  0.00308461  0.00311907  0.00316516  0.00359788  0.00308457  0.00391964  0.00310506  0.00449182  0.00303605  0.00440325  0.00419154  0.00546031  0.00325813  0.00304345  0.00305032  0.00668673  0.00303483  0.00355589  0.00307022  0.00311635  0.00352806  0.00336834  0.00549884  0.00311609  0.00580219  0.00918496  0.00311875  0.00315406  0.00359305  0.00302582  0.00506585  0.00338983  0.00372613  0.00309951  0.00303977  0.00407148  0.00313037  0.00308215  0.00493484  0.00312467  0.0037742  0.0030674  0.00409088  0.00372774  0.00310188  0.00302891  0.00309117  0.00415058  0.00320078  0.00459853  0.00575757  0.00305291  0.00350346  0.00330311  0.00324831  0.00304065  0.00375381  0.00398884  0.00303737  0.00309866  0.00305026  0.00319841  0.00355204  0.00431166  0.00307848  0.00303207  0.0034046  0.00316849  0.00305071  0.00649583  0.00331968  0.00304832  0.00410324  0.00308788  0.00308481  0.00347125  0.00309039  0.00406259  0.00396178  0.00392537  0.0032265  0.00358993  0.00343021  0.0035079  0.00305313  0.00357005  0.0043272  0.00427092  0.00305022  0.00344068  0.00305549  0.00327075  0.00523292  0.0127906 | 4.00E-09  3.40E-09  7.70E-09  3.80E-09  2.70E-09  4.60E-08  4.00E-11  5.50E-10  1.30E-60  4.30E-11  7.20E-11  1.30E-08  3.40E-08  3.40E-08  6.30E-12  9.10E-13  2.80E-08  3.80E-12  1.40E-08  1.60E-12  7.70E-15  4.90E-09  2.30E-09  3.20E-08  1.60E-15  1.00E-44  8.40E-14  1.40E-178  4.90E-10  6.60E-09  1.90E-08  8.30E-10  9.90E-22  3.80E-08  3.80E-08  1.10E-14  2.70E-10  3.10E-08  1.80E-09  3.50E-10  2.40E-09  2.90E-16  1.40E-11  4.60E-10  2.10E-10  3.10E-08  2.30E-12  2.60E-08  1.40E-09  4.60E-08  2.40E-14  1.70E-08  2.50E-08  4.90E-08  6.20E-09  6.90E-10  9.30E-09  9.90E-09  2.30E-11  2.00E-08  8.70E-15  6.70E-09  6.80E-09  8.40E-09  5.60E-09  3.80E-08  5.10E-18  2.50E-08  3.70E-10  2.70E-09  4.00E-09  1.40E-09  3.10E-09  2.90E-08  6.40E-12  1.40E-17  1.00E-10  1.70E-11  7.30E-30  1.80E-08  1.90E-09  2.70E-10  2.30E-09  9.70E-09  2.90E-13  3.40E-09  7.60E-22  1.40E-08  1.60E-09  5.90E-09  1.60E-10  2.80E-08  6.70E-13  2.40E-09  1.20E-08  3.60E-08 | 0.70882  0.565333  0.40423  0.605114  0.361153  0.232457  0.585731  0.184023  0.615206  0.134041  0.470852  0.13763  0.154942  0.089115  0.679886  0.557689  0.435133  0.054327  0.468553  0.237423  0.429053  0.383186  0.246133  0.280364  0.083483  0.604193  0.074582  0.97277  0.61881  0.626705  0.230728  0.490369  0.102253  0.276349  0.209454  0.420617  0.462259  0.16931  0.372967  0.405493  0.10497  0.381706  0.202827  0.428653  0.164488  0.208646  0.400576  0.510245  0.604293  0.157151  0.369127  0.124072  0.074515  0.437454  0.24819  0.32785  0.680585  0.505254  0.206155  0.823865  0.454624  0.392206  0.451772  0.339998  0.759525  0.147849  0.410052  0.528054  0.720352  0.354749  0.463343  0.057704  0.336989  0.446061  0.162767  0.401541  0.403447  0.737192  0.597353  0.168868  0.176998  0.182509  0.674892  0.239585  0.262687  0.247918  0.459939  0.40703  0.145599  0.153147  0.539592  0.735045  0.542982  0.31415  0.093465  0.0142 | 0.00016  0.00016  0.00015  0.00016  0.00016  0.00014  0.00020  0.00018  0.00123  0.00020  0.00020  0.00015  0.00014  0.00015  0.00022  0.00023  0.00014  0.00022  0.00015  0.00023  0.00028  0.00016  0.00016  0.00014  0.00029  0.00091  0.00026  0.00363  0.00018  0.00016  0.00014  0.00017  0.00043  0.00014  0.00014  0.00028  0.00018  0.00014  0.00017  0.00018  0.00016  0.00031  0.00021  0.00018  0.00019  0.00014  0.00023  0.00014  0.00017  0.00014  0.00028  0.00015  0.00014  0.00014  0.00015  0.00018  0.00015  0.00015  0.00021  0.00015  0.00028  0.00015  0.00015  0.00015  0.00016  0.00014  0.00034  0.00014  0.00018  0.00016  0.00016  0.00017  0.00017  0.00014  0.00022  0.00033  0.00019  0.00021  0.00059  0.00015  0.00016  0.00018  0.00016  0.00015  0.00024  0.00016  0.00043  0.00020  0.00017  0.00016  0.00019  0.00014  0.00024  0.00016  0.00015  0.00014 | 0.76  0.76  0.71  0.75  0.76  0.64  0.94  0.83  5.76  0.95  0.91  0.70  0.65  0.69  1.02  1.09  0.66  1.03  0.69  1.07  1.30  0.73  0.77  0.65  1.37  4.27  1.21  17.00  0.83  0.73  0.68  0.81  2.02  0.65  0.65  1.31  0.85  0.67  0.77  0.84  0.76  1.44  0.98  0.84  0.87  0.66  1.06  0.66  0.78  0.64  1.30  0.68  0.66  0.64  0.72  0.85  0.71  0.71  0.96  0.68  1.29  0.72  0.72  0.71  0.73  0.66  1.60  0.66  0.86  0.76  0.75  0.79  0.81  0.66  1.01  1.56  0.89  0.99  2.77  0.69  0.77  0.86  0.76  0.72  1.13  0.75  2.00  0.93  0.79  0.75  0.88  0.66  1.12  0.77  0.70  0.65 | 109.95 |
| **Smoking initiation** | rs3001723  rs7555507  rs6669839  rs12042107  rs301805  rs12025237  rs2050586  rs2046850  rs6728726  rs78411160  rs6433897  rs266047  rs4674993  rs578584  rs35702515  rs13030994  rs12474587  rs2107300  rs1445649  rs6788098  rs12632110  rs11712680  rs1154693  rs66680800  rs1869243  rs9835772  rs962625  rs993700  rs13145728  rs10001365  rs6893752  rs12186738  rs1385108  rs4044321  rs4352629  rs72789632  rs9401770  rs222449  rs3800227  rs10498846  rs240963  rs12333760  rs10233018  rs10279261  rs10260968  rs12112638  rs4236259  rs2140114  rs3801289  rs1565735  rs1899896  rs13261666  rs12545053  rs2631024  rs4543592  rs2378662  rs10114490  rs10905461  rs12356821  rs10159545  rs9423279  rs7938812  rs6265  rs7929518  rs4523689  rs11057005  rs4759228  rs7969559  rs1971318  rs7322872  rs3904512  rs76214862  rs12441907  rs1435741  rs4785836  rs7197072  rs1050847  rs4781977  rs11078713  rs7224742  rs11658881  rs11872397  rs72896886  rs76608582  rs1555445  rs117143374  rs134529 | A  T  T  C  G  C  C  T  C  C  C  A  G  T  T  A  T  G  C  T  G  C  G  T  C  T  G  C  C  A  G  T  T  G  T  T  A  T  G  T  C  C  G  A  A  G  G  T  C  A  T  T  G  G  C  A  A  C  C  G  G  G  T  G  G  G  C  G  T  T  A  C  A  A  C  T  T  C  G  T  G  A  C  A  T  C  C | G  C  C  T  T  A  G  C  T  A  T  G  A  A  G  G  G  C  T  A  A  A  A  G  T  A  A  T  G  G  A  G  C  A  C  C  G  A  A  C  T  T  A  G  G  A  T  C  A  T  C  G  A  A  T  G  G  T  G  C  C  T  C  A  A  A  G  A  C  C  G  A  C  G  T  C  C  T  A  C  A  G  G  C  A  T  T | 0.03351  -0.02414  0.02600  -0.02228  0.02147  -0.03300  -0.02055  -0.02481  0.03545  0.02054  0.02245  -0.03051  -0.02521  0.02868  0.02524  0.03609  0.02763  -0.02720  0.02399  -0.03135  -0.02338  -0.02705  0.03262  -0.02027  0.01974  0.02405  0.02372  -0.02593  -0.02325  -0.02499  -0.02410  -0.03326  0.02466  -0.02784  -0.02753  -0.03289  0.02773  -0.02532  0.02281  0.02061  -0.04104  -0.02905  0.02707  -0.02142  -0.02032  -0.02453  -0.02477  -0.02326  -0.02206  -0.03762  0.02645  -0.02689  0.02028  -0.02296  0.02193  0.02095  -0.02551  -0.02396  0.03937  0.02625  -0.02051  0.04379  -0.03179  0.02424  -0.02061  -0.02093  -0.02169  -0.02438  0.02851  -0.02557  -0.02116  -0.02499  -0.02921  0.02942  -0.02047  -0.02477  -0.02162  -0.02387  -0.02017  -0.02071  0.02014  -0.02477  -0.02689  -0.04956  0.02255  0.02929  -0.01998 | 0.0038983  0.00355604  0.0043955  0.0035682  0.00361329  0.00533915  0.00370828  0.00447842  0.00473279  0.00365894  0.00405809  0.00373855  0.00443619  0.00359627  0.00423093  0.0035563  0.00358234  0.00492533  0.00356484  0.00368905  0.00375292  0.00457843  0.00491232  0.00365268  0.00356289  0.00414237  0.00403803  0.00429163  0.00366263  0.00364155  0.00407356  0.00502051  0.00415673  0.00371058  0.00356881  0.00528628  0.003986  0.00442796  0.00405809  0.00355561  0.00483712  0.00480127  0.00355741  0.00366263  0.00360938  0.00404299  0.00355661  0.00373402  0.00373982  0.0044613  0.00388691  0.00355604  0.00363668  0.00402823  0.00356244  0.00356645  0.00453171  0.00414505  0.0050491  0.00372727  0.00370828  0.00363668  0.00457843  0.00428466  0.00364321  0.00357892  0.00393413  0.00395946  0.00492533  0.00433474  0.0035765  0.00454745  0.00452262  0.00359095  0.00365894  0.0041686  0.00358893  0.00436473  0.00360561  0.00365532  0.00361066  0.00409477  0.00483712  0.00825958  0.0038234  0.00526909  0.00366078 | 8.12E-18  1.14E-11  3.36E-09  4.22E-10  2.80E-09  6.52E-10  3.00E-08  3.03E-08  6.73E-14  2.03E-08  3.16E-08  3.36E-16  1.32E-08  1.50E-15  2.43E-09  3.56E-24  1.25E-14  3.27E-08  1.68E-11  1.91E-17  4.78E-10  3.51E-09  3.12E-11  2.83E-08  2.97E-08  6.32E-09  4.37E-09  1.53E-09  2.14E-10  6.65E-12  3.25E-09  3.42E-11  3.00E-09  6.08E-14  1.22E-14  5.02E-10  3.47E-12  1.08E-08  1.93E-08  6.62E-09  2.16E-17  1.44E-09  2.75E-14  5.00E-09  1.75E-08  1.34E-09  3.35E-12  4.70E-10  3.74E-09  3.42E-17  1.04E-11  3.90E-14  2.43E-08  1.18E-08  7.46E-10  4.16E-09  1.81E-08  7.35E-09  6.27E-15  1.84E-12  3.21E-08  2.71E-33  3.77E-12  1.56E-08  1.55E-08  4.85E-09  3.58E-08  7.31E-10  7.06E-09  3.58E-09  3.23E-09  3.99E-08  1.06E-10  2.64E-16  2.26E-08  2.77E-09  1.67E-09  4.54E-08  2.23E-08  1.43E-08  2.43E-08  1.43E-09  2.75E-08  1.94E-09  3.65E-09  2.76E-08  4.85E-08 | 0.321  0.496  0.204  0.527  0.559  0.124  0.355  0.187  0.829  0.631  0.754  0.529  0.207  0.605  0.162  0.485  0.404  0.845  0.525  0.623  0.647  0.174  0.856  0.397  0.481  0.235  0.24  0.766  0.358  0.405  0.766  0.154  0.239  0.642  0.492  0.12  0.273  0.793  0.701  0.473  0.836  0.204  0.503  0.619  0.597  0.275  0.499  0.518  0.351  0.212  0.286  0.522  0.397  0.737  0.468  0.556  0.198  0.718  0.14  0.375  0.641  0.424  0.203  0.765  0.408  0.43  0.27  0.688  0.141  0.782  0.429  0.202  0.186  0.425  0.398  0.238  0.505  0.205  0.454  0.595  0.418  0.252  0.144  0.0389  0.337  0.12  0.349 | 0.00049  0.00029  0.00022  0.00025  0.00023  0.00024  0.00019  0.00019  0.00036  0.00020  0.00019  0.00046  0.00021  0.00039  0.00017  0.00065  0.00037  0.00019  0.00029  0.00046  0.00025  0.00021  0.00026  0.00020  0.00019  0.00021  0.00021  0.00024  0.00025  0.00030  0.00021  0.00029  0.00022  0.00036  0.00038  0.00023  0.00031  0.00021  0.00022  0.00021  0.00046  0.00027  0.00037  0.00022  0.00020  0.00024  0.00031  0.00027  0.00022  0.00047  0.00029  0.00036  0.00020  0.00020  0.00024  0.00022  0.00021  0.00023  0.00037  0.00032  0.00019  0.00094  0.00033  0.00021  0.00021  0.00021  0.00019  0.00026  0.00020  0.00022  0.00022  0.00020  0.00026  0.00042  0.00020  0.00022  0.00023  0.00019  0.00020  0.00021  0.00020  0.00023  0.00018  0.00018  0.00023  0.00018  0.00018 | 3.33  1.98  1.49  1.68  1.55  1.61  1.32  1.27  2.42  1.34  1.27  3.16  1.42  2.68  1.18  4.43  2.50  1.32  1.95  3.14  1.70  1.43  1.79  1.34  1.32  1.41  1.40  1.64  1.69  2.05  1.42  1.96  1.51  2.43  2.58  1.55  2.08  1.43  1.48  1.44  3.14  1.86  2.49  1.47  1.35  1.63  2.09  1.84  1.51  3.22  1.94  2.46  1.34  1.39  1.63  1.47  1.41  1.58  2.54  2.20  1.32  6.38  2.22  1.44  1.40  1.46  1.26  1.74  1.34  1.52  1.49  1.37  1.76  2.88  1.37  1.51  1.59  1.26  1.37  1.41  1.34  1.57  1.21  1.25  1.55  1.23  1.23 | 158.72 |

**Supplementary Table S2.** The statistical power results of GERD and lung cancer.

| **Outcomes** | **Sample size** | **α** | **K** | **OR** | **R2** | **Power** | **Non-Centrality-Parameter** |
| --- | --- | --- | --- | --- | --- | --- | --- |
| **Lung cancer** | 85,716 | 0.05 | 0.341 | 1.50 | 0.035 | 1 | 127.91 |
| **Squamous cell lung carcinoma** | 63,053 | 0.05 | 0.118 | 1.31 | 0.035 | 1 | 20.73 |
| **Lung adenocarcinoma** | 66,756 | 0.05 | 0.169 | 1.21 | 0.034 | 0.95 | 13.19 |
| **Small cell lung carcinoma** | 24,108 | 0.05 | 0.111 | 1.72 | 0.032 | 1 | 35.39 |

**Supplementary Table S3.** Outliers selected by MR-PRESSO.

| **Outcomes** | **No** | **SNP (outliers)** | **Outliers p-value** | **Beta. exposure** | **Beta. outcome** | **Se. exposure** | **Se. outcome** | **Effect allele** | **Other allele** |
| --- | --- | --- | --- | --- | --- | --- | --- | --- | --- |
| **Lung cancer** | 1 | rs1592757 | <0.015 | 0.031 | -0.041 | 0.005 | 0.012 | C | G |
|  | 2 | rs329122 | 0.015 | -0.029 | -0.051 | 0.005 | 0.012 | A | G |
|  | 3 | rs7032155 | <0.015 | 0.028 | -0.039 | 0.005 | 0.012 | A | C |
|  | 4 | rs6711584 | 0.210 | 0.032 | 0.044 | 0.005 | 0.012 | A | G |
|  | 5 | rs773109 | 0.840 | -0.038 | -0.044 | 0.005 | 0.013 | A | G |
|  | 6 | rs215614 | 0.450 | -0.033 | -0.043 | 0.005 | 0.012 | A | G |
|  | 7 | rs2782641 | 0.375 | 0.027 | 0.042 | 0.005 | 0.012 | A | G |
| **Lung Adenocarcinoma** | 1 | rs1592757 | 0.015 | 0.031 | -0.054 | 0.005 | 0.017 | C | G |
|  | 2 | rs6711584 | <0.015 | 0.032 | 0.067 | 0.005 | 0.016 | A | G |
|  | 3 | rs4382592 | 0.526 | -0.030 | -0.053 | 0.005 | 0.017 | G | T |
|  | 4 | rs7032155 | 0.745 | 0.028 | -0.038 | 0.005 | 0.017 | A | C |
| **Small cell lung carcinoma** | 1 | rs2815749 | 0.281 | 0.039 | -0.091 | 0.006 | 0.039 | G | A |

**Supplementary Table S4.** Genetic instruments from the Phenoscanner database associated with smoking and alcohol.

| **No** | **SNP** | **Hg19_coordinates** | **Effect allele** | **Other allele** | **Trait** | **Ancestry** | **Beta** | **SE** | **p-value** | **n** |
| --- | --- | --- | --- | --- | --- | --- | --- | --- | --- | --- |
| 1 | rs6711584 | chr2:104421692 | A | G | Past tobacco smoking | European | -0.01759 | 0.003193 | 3.59E-08 | 310749 |
| 2 | rs215614 | chr7:32347335 | A | G | Pack years of smoking preview only | European | -0.0294 | 0.004517 | 7.63E-11 | 101726 |
| 3 | rs2240326 | chr3:50128386 | G | A | Alcohol intake frequency | European | 0.02225 | 0.003554 | 3.82E-10 | 336965 |
| 4 | rs7685686 | chr4:3207142 | A | G | Alcohol intake frequency | European | 0.02012 | 0.0036 | 2.29E-08 | 336965 |
| 5 | rs13107325 | chr4:103188709 | T | C | Alcohol intake frequency | European | 0.05053 | 0.006762 | 7.88E-14 | 336965 |
| 6 | rs9372625 | chr6:98344031 | A | G | Alcohol intake frequency | European | -0.0277 | 0.003668 | 4.33E-14 | 336965 |
| 7 | rs9940128 | chr16:53800754 | A | G | Alcohol intake frequency | European | 0.02098 | 0.003598 | 5.52E-09 | 336965 |
| 8 | rs903959 | chr8:142630782 | A | T | Alcohol intake frequency | European | 0.02166 | 0.003633 | 2.49E-09 | 336965 |
| 9 | rs2815749 | chr1:72814783 | G | A | Body mass index adjusted for smoking | European | 0.0331 | 0.0047 | 1.70E-12 | 186004 |
| 10 | rs903678 | chr1:201809918 | A | G | Body mass index adjusted for smoking | European | 0.0243 | 0.0042 | 6.09E-09 | 158040 |
| 11 | rs2744961 | chr6:34655000 | T | C | Body mass index adjusted for smoking | European | 0.0229 | 0.0041 | 2.79E-08 | 163844 |
