# Supplementary material for: The role of smoking and alcohol in mediating the effect of gastroesophageal reflux disease on lung cancer: A Mendelian randomization study
Source: Front Genet. 2023 Jan 16;13:1054132. doi: 10.3389/fgene.2022.1054132 (PMC9885128; doi:10.3389/fgene.2022.1054132)
Supplement: Supplementary file 2 [file DataSheet1.docx]

**Supplementary Figure S1.** Genetically predicted associations of GERD with lung cancer and subgroups.

Genetically predicted association of GERD with lung cancer.

(A) Scatter plot

(B) funnel plot

(C) leave-one-out plot


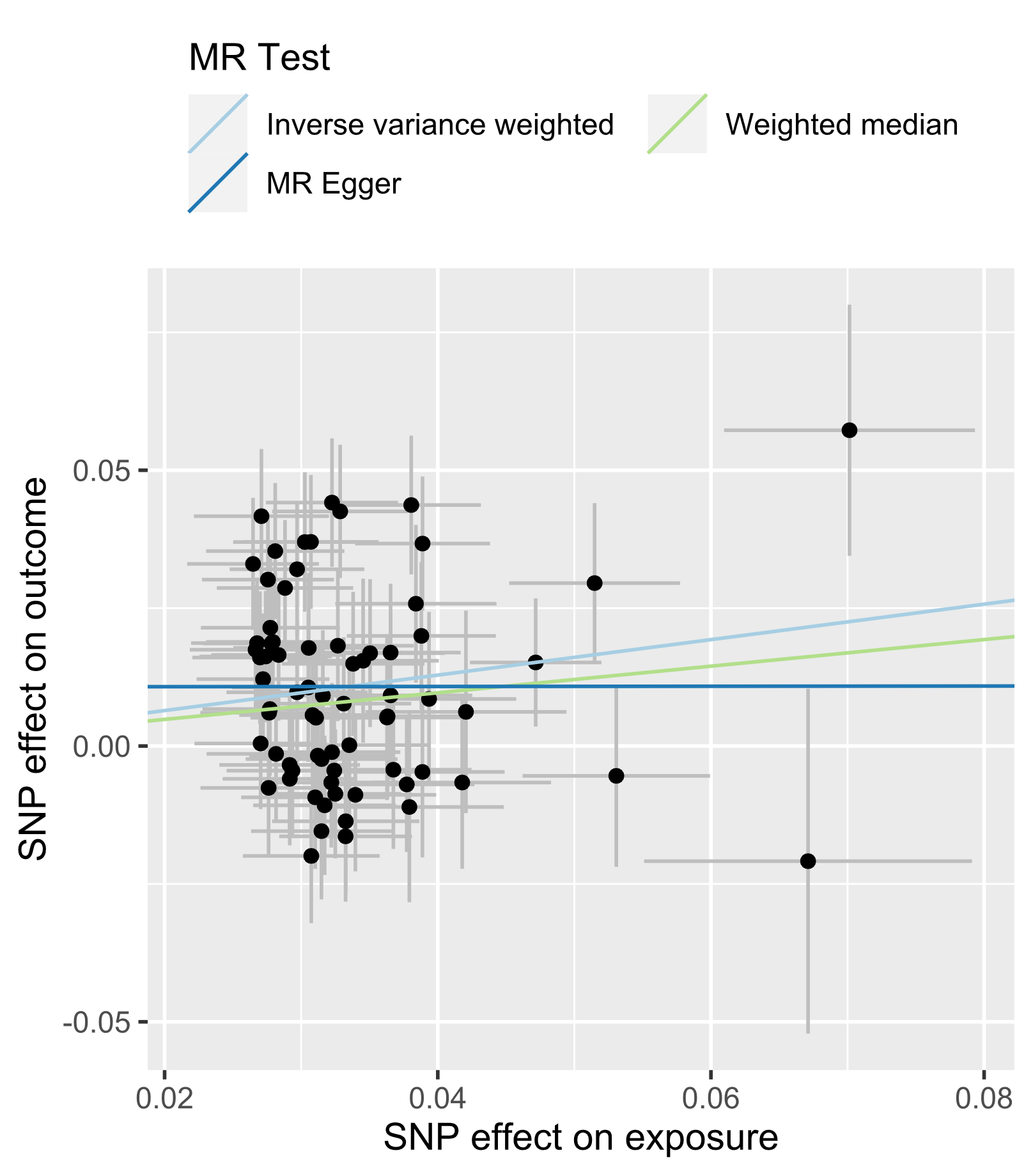

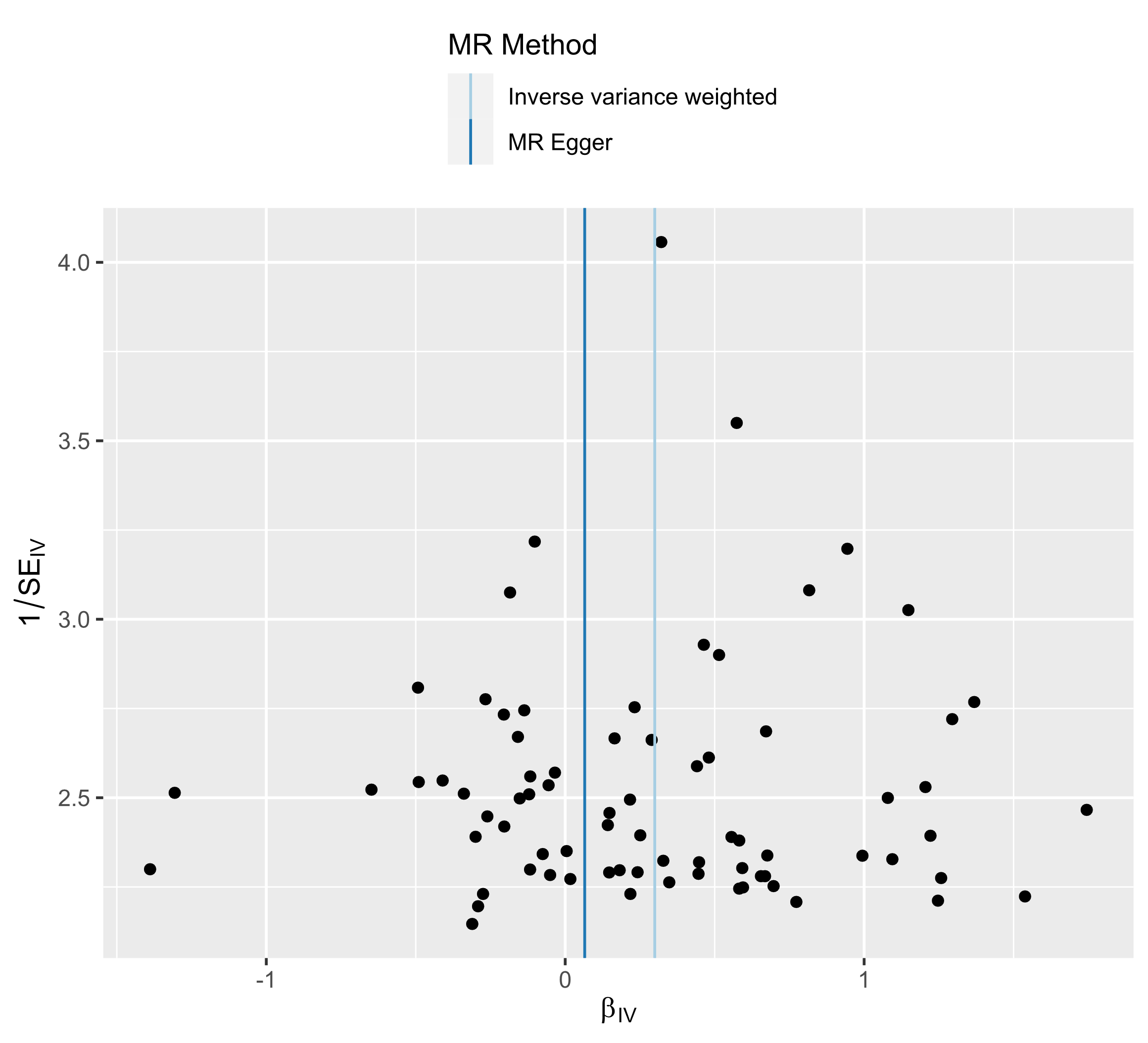


(A) (B)


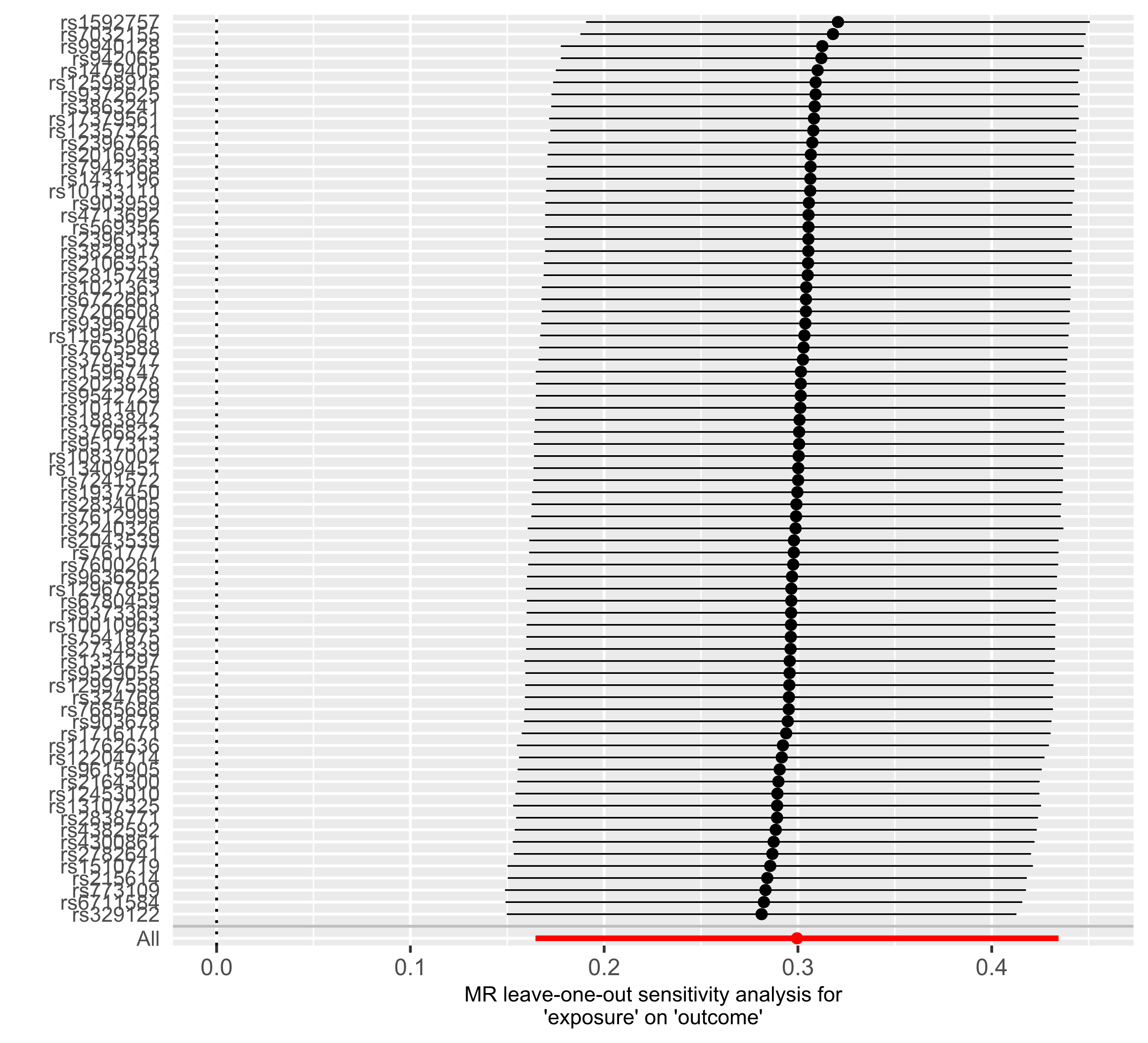


(C)

Genetically predicted association of GERD with squamous cell lung cancer.

(A) Scatter plot

(B) funnel plot

(C) leave-one-out plot


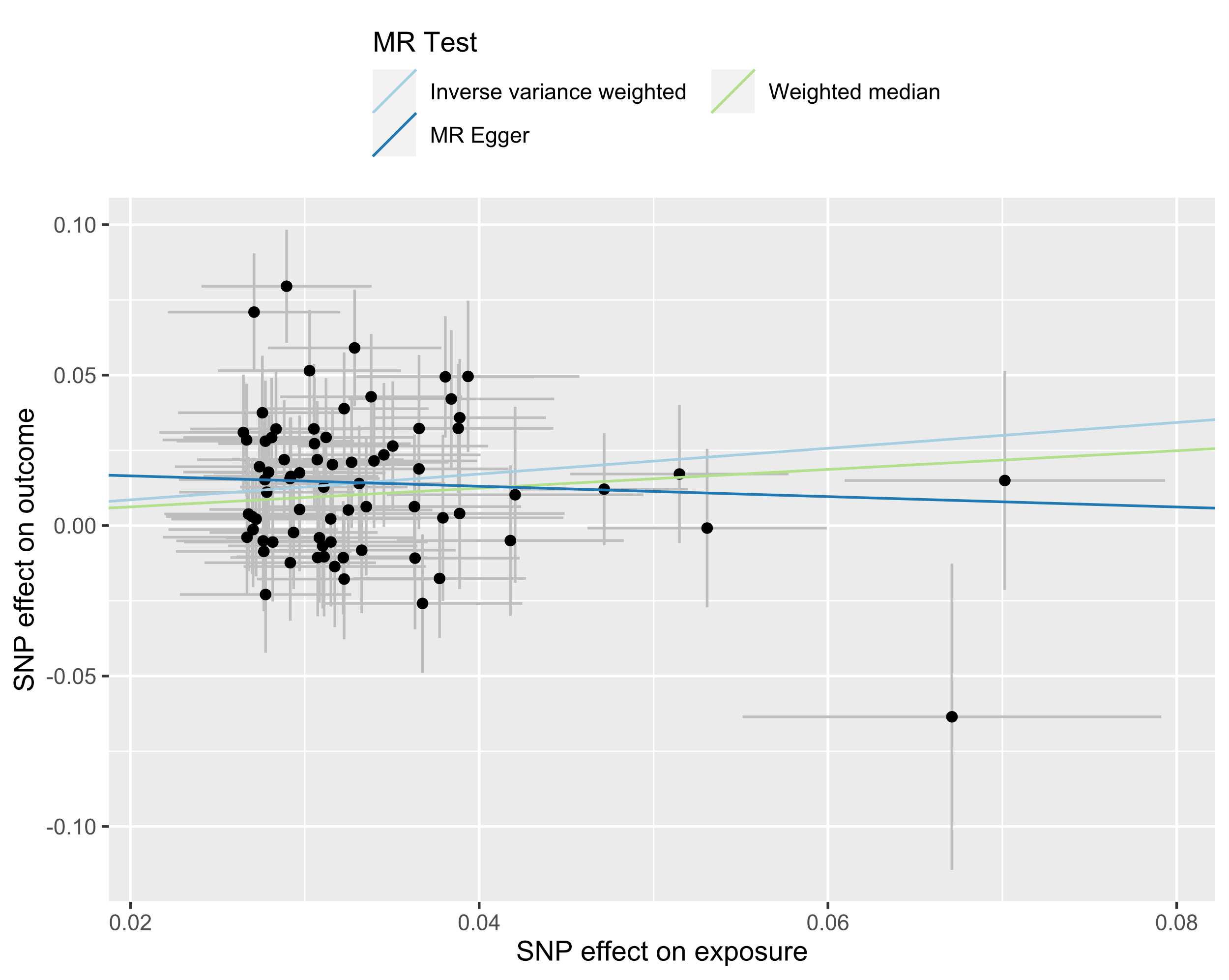

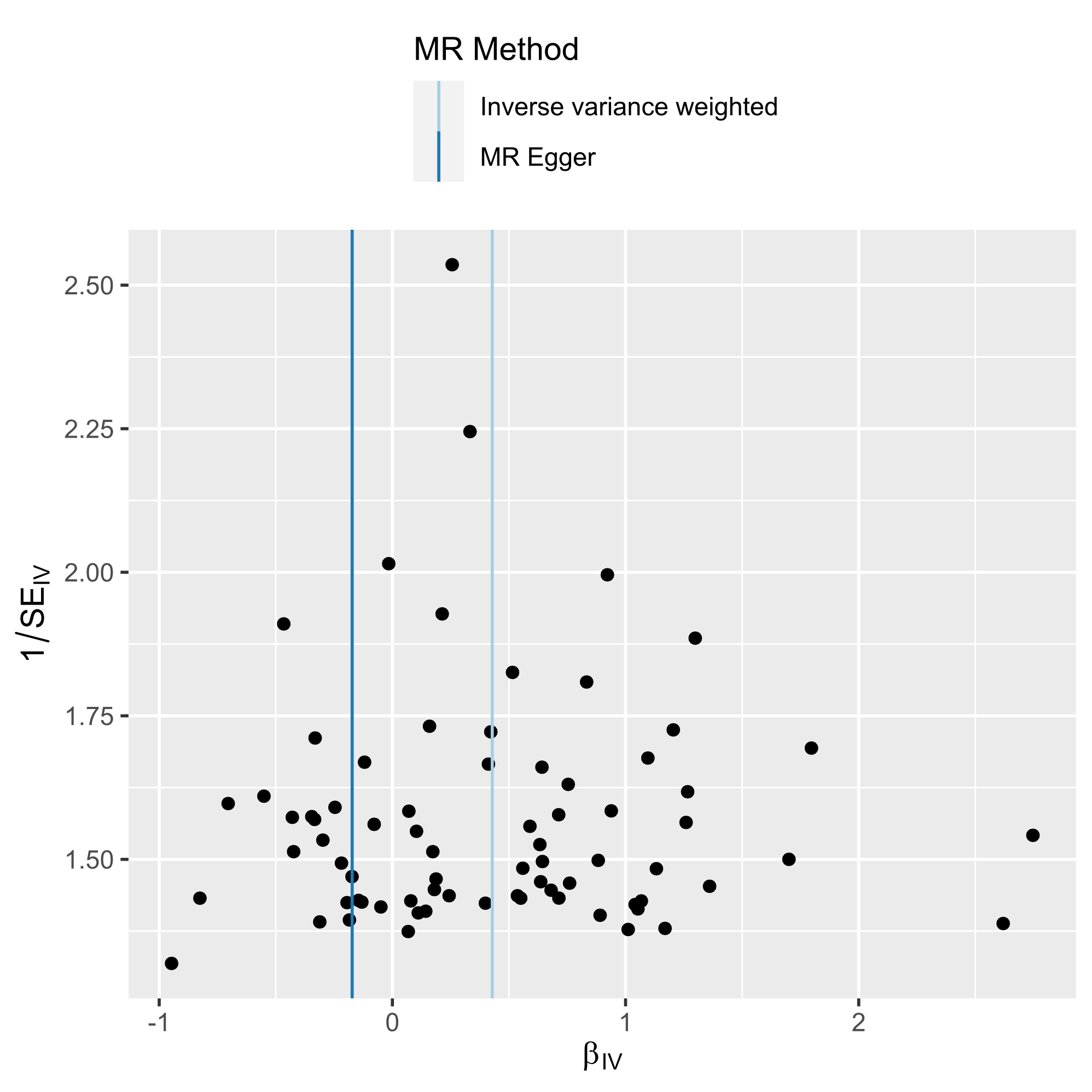


(A) (B)


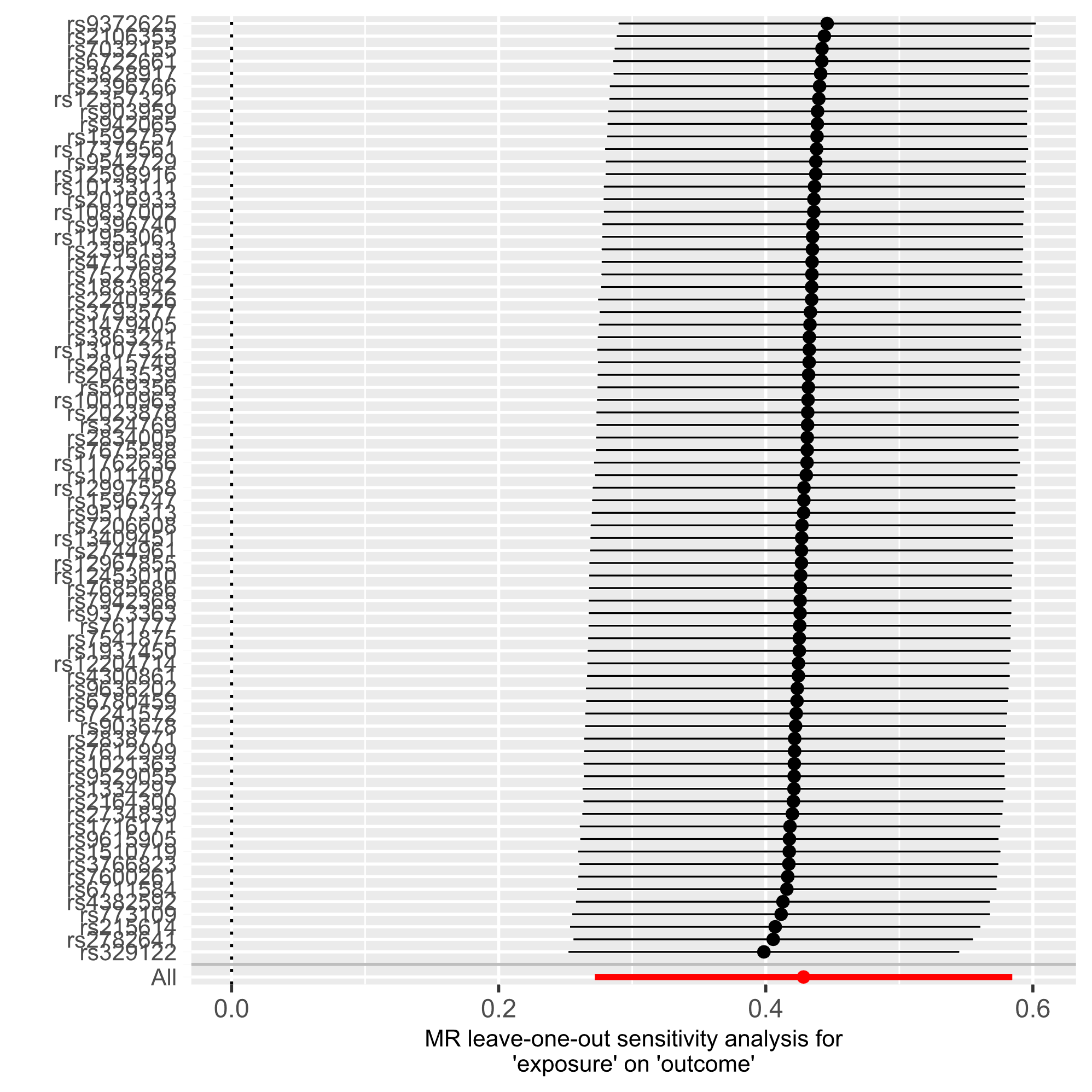


(C)

Genetically predicted association of GERD with lung adenocarcinoma.

(A) Scatter plot

(B) funnel plot

(C) leave-one-out plot


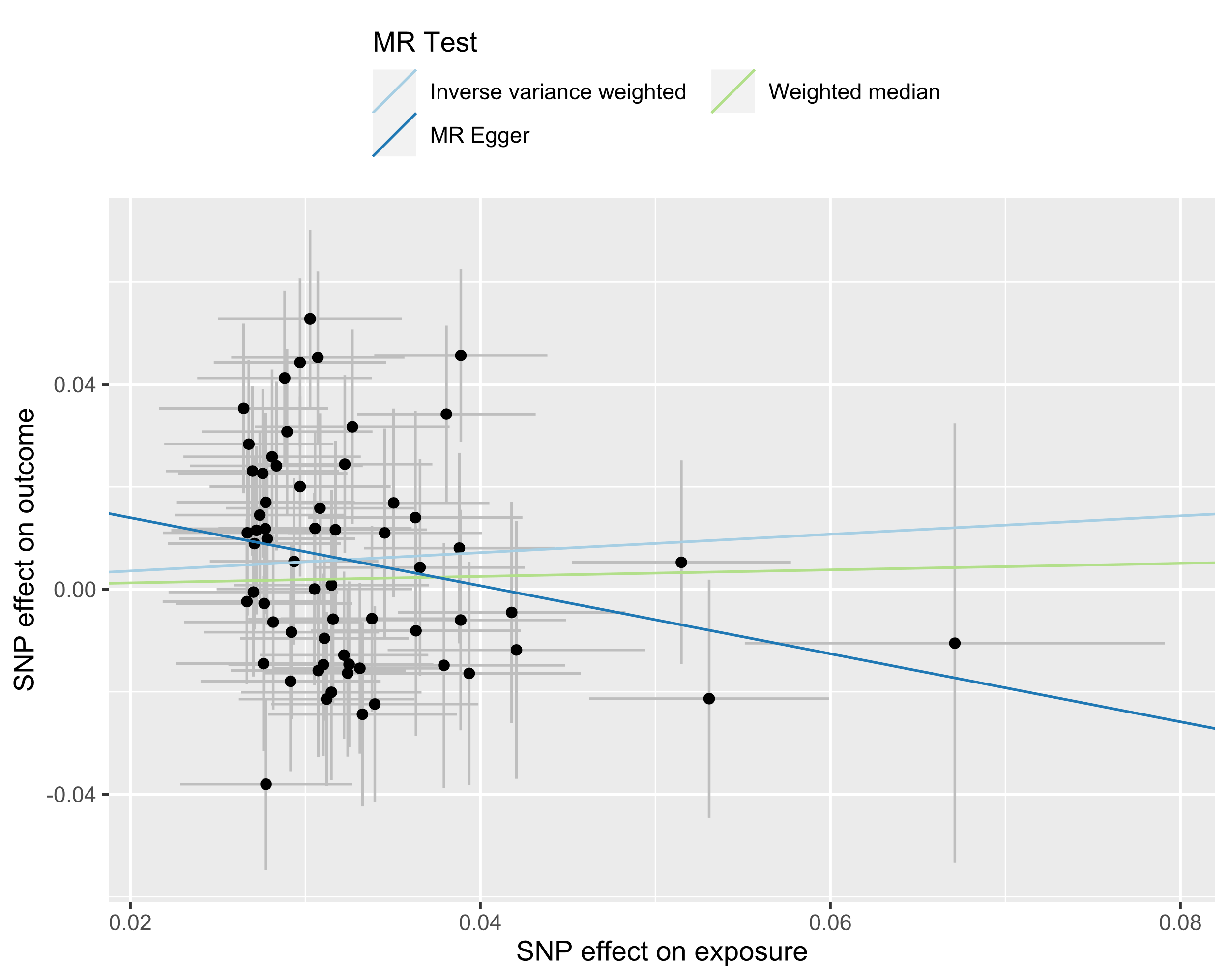

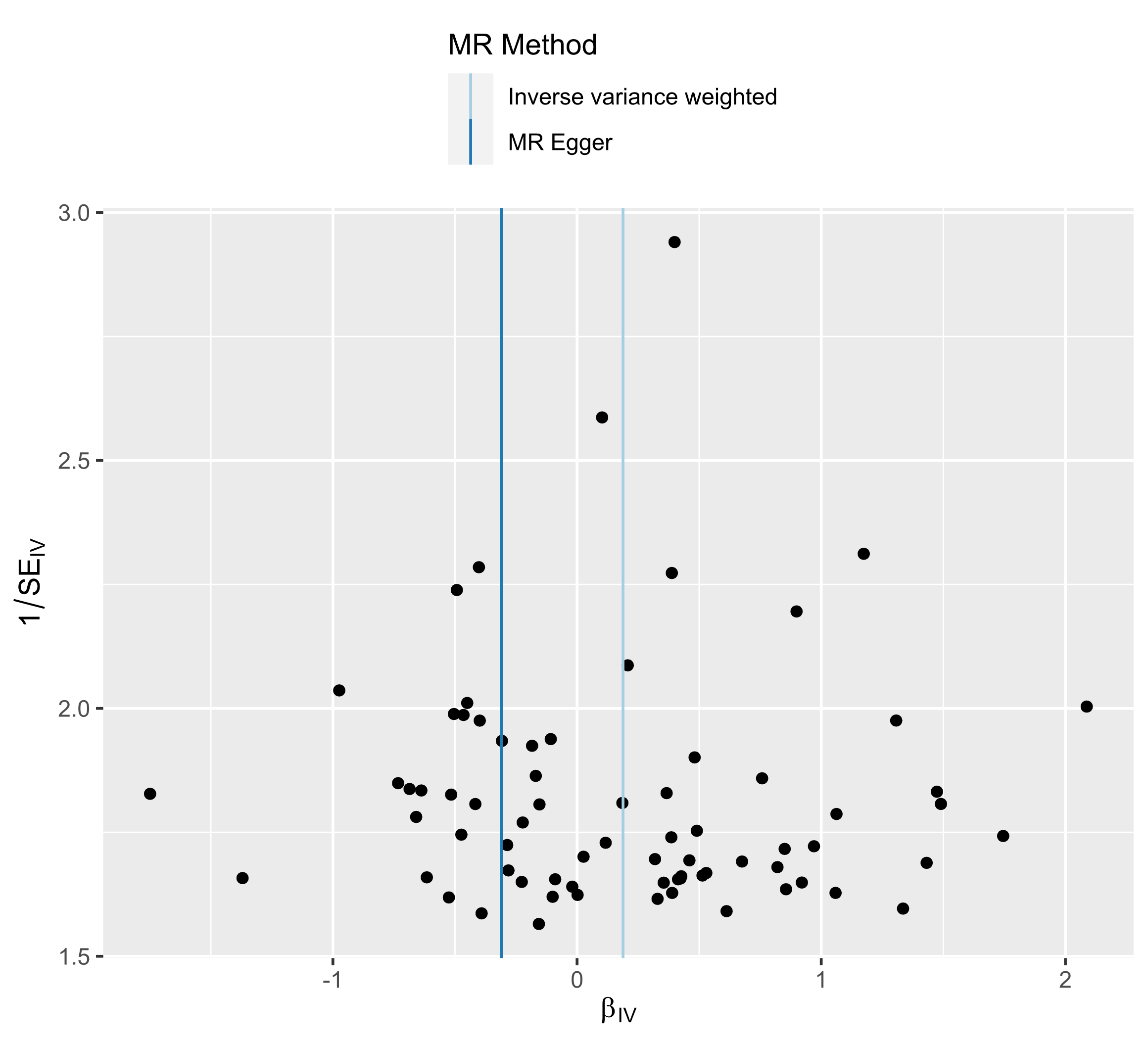


(A) (B)


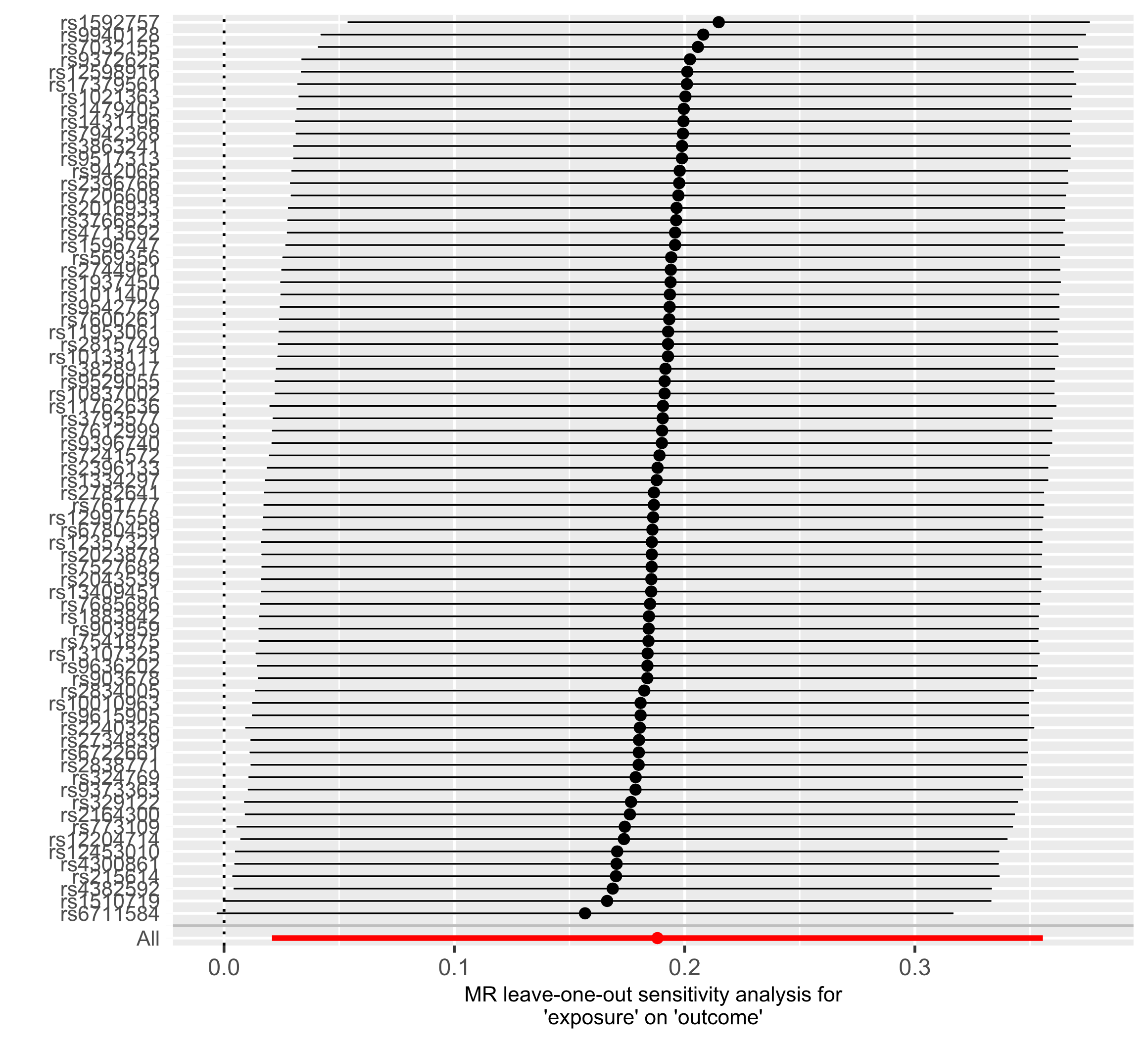


(C)

Genetically predicted association of GERD with small cell lung cancer

(A) Scatter plot

(B) funnel plot

(C) leave-one-out plot


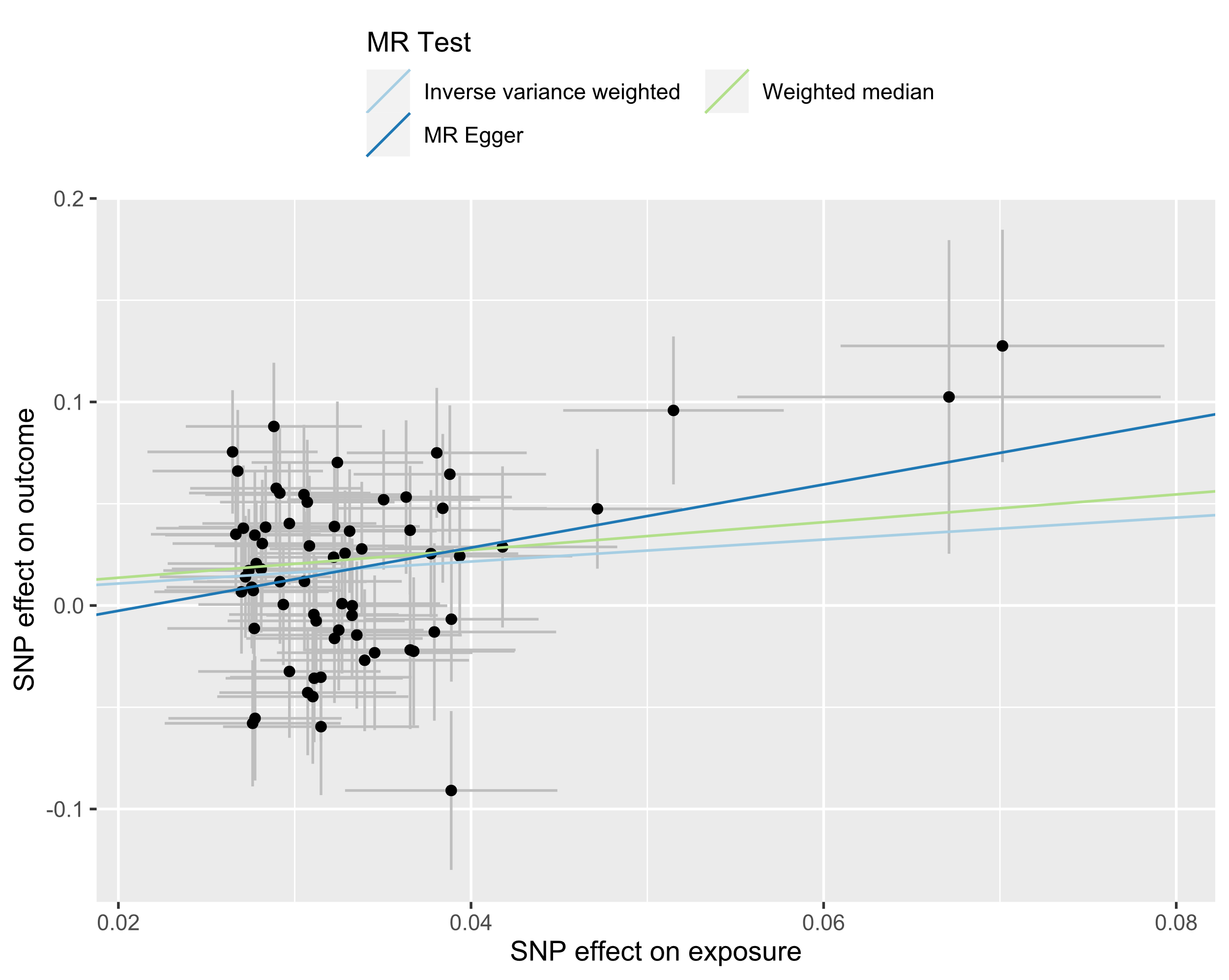

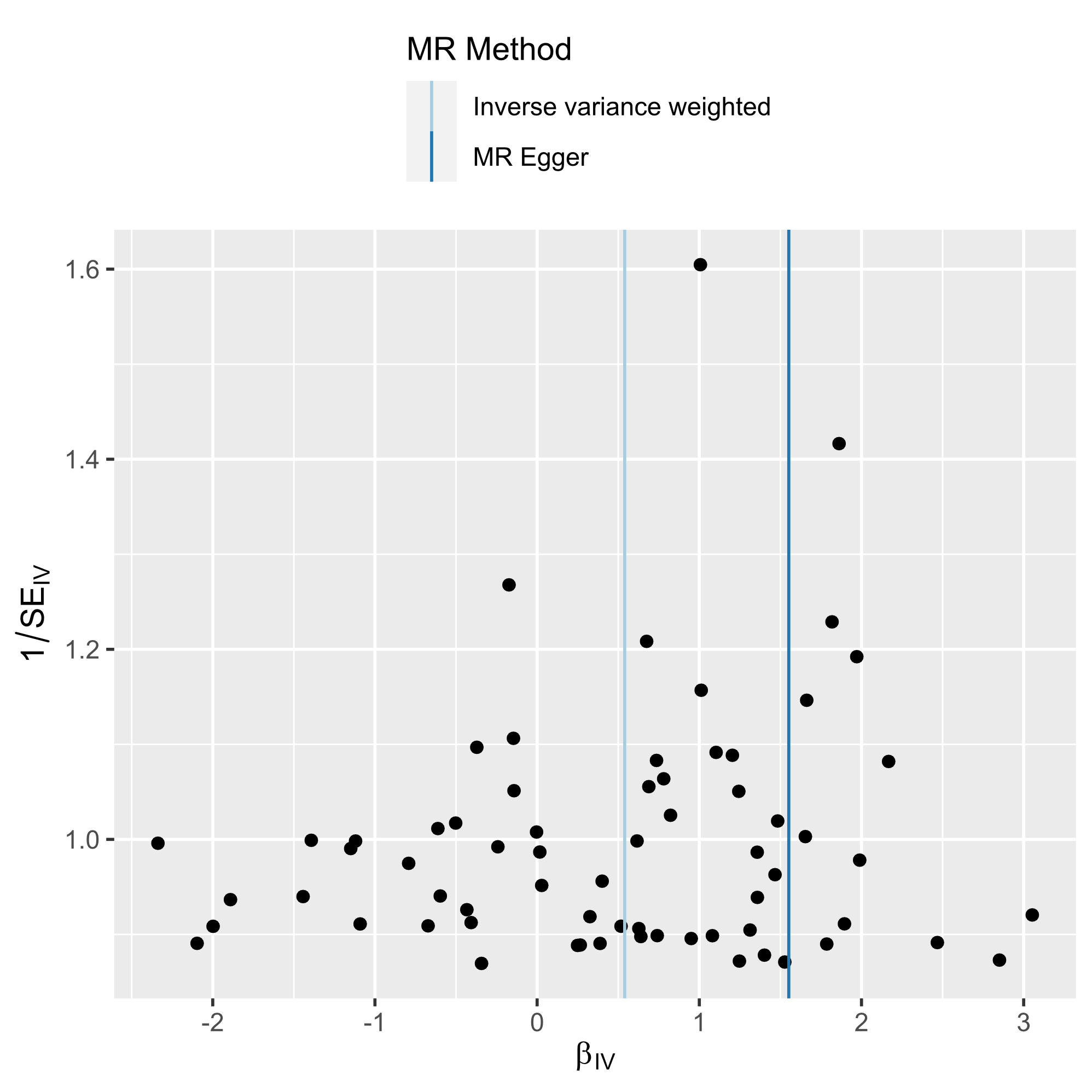


(A) (B)


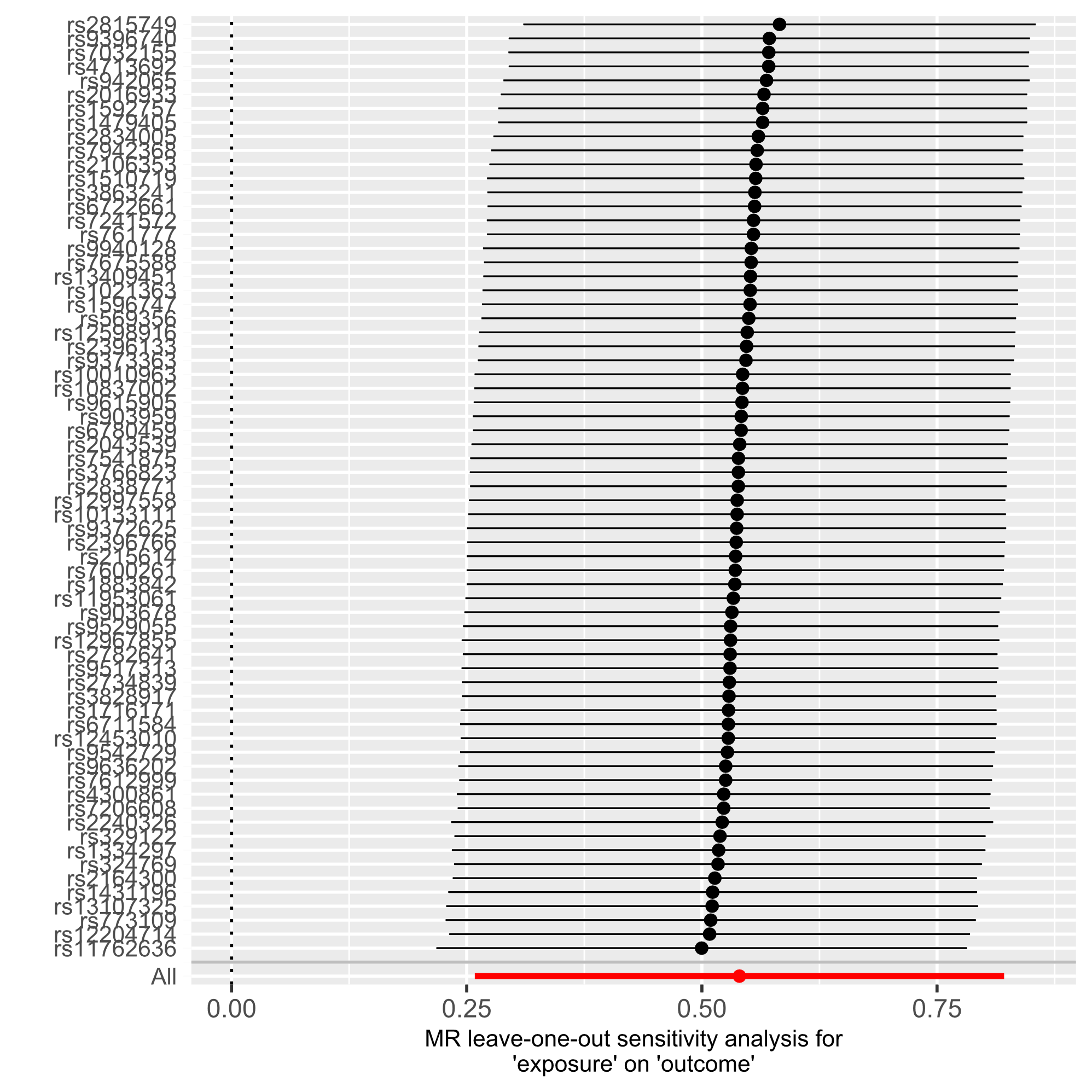


(C)
